# Genetic analysis of heat tolerance in hot pepper: insights from comprehensive phenotyping and QTL mapping

**DOI:** 10.3389/fpls.2023.1232800

**Published:** 2023-08-25

**Authors:** Aruna TS, Arpita Srivastava, Bhoopal Singh Tomar, Tusar Kanti Behera, Hari Krishna, Pradeep Kumar Jain, Renu Pandey, Bhupinder Singh, Ruchi Gupta, Manisha Mangal

**Affiliations:** ^1^ Division of Vegetable Science, Indian Agricultural Research Institute, Indian Council of Agricultural Research (ICAR), New Delhi, India; ^2^ Indian Council of Agricultural Research-Indian Institute of Vegetable Research (IIVR), Indian Council of Agricultural Research (ICAR), Varanasi, India; ^3^ Division of Genetics, Indian Agricultural Research Institute, Indian Council of Agricultural Research (ICAR), New Delhi, India; ^4^ National Institute of Plant Biotechnology, Indian Council of Agricultural Research (ICAR), New Delhi, India; ^5^ Division of Plant Physiology, Indian Agricultural Research Institute, Indian Council of Agricultural Research (ICAR), New Delhi, India; ^6^ Division of Environment Science, Indian Agricultural Research Institute, Indian Council of Agricultural Research (ICAR), New Delhi, India; ^7^ Department of Computer Sciences, Jamia Milia Islamia, New Delhi, India

**Keywords:** *Capsicum annuum*, heat tolerance, QTL mapping, additive effect, dominance effect, phenotyping, genotyping

## Abstract

High temperatures present a formidable challenge to the cultivation of hot pepper, profoundly impacting not only vegetative growth but also leading to flower and fruit abscission, thereby causing a significant reduction in yield. To unravel the intricate genetic mechanisms governing heat tolerance in hot pepper, an F_2_ population was developed through the crossing of two distinct genotypes exhibiting contrasting heat tolerance characteristics: DLS-161-1 (heat tolerant) and DChBL-240 (heat susceptible). The F_2_ population, along with the parental lines, was subjected to comprehensive phenotyping encompassing diverse morphological, physiological, and biochemical heat-related traits under high temperature conditions (with maximum temperature ranging from 31 to 46.5°C and minimum temperature from 15.4 to 30.5°C). Leveraging the Illumina Nova Seq-6000 platform, Double digest restriction-site associated DNA sequencing (ddRAD-seq) was employed to generate 67.215 Gb data, with subsequent alignment of 218.93 million processed reads against the reference genome of *Capsicum annuum*. Subsequent variant calling and ordering resulted in 5806 polymorphic SNP markers grouped into 12 LGs. Further QTL analysis identified 64 QTLs with LOD values ranging from 2.517 to 11.170 and explained phenotypic variance ranging from 4.05 to 19.39%. Among them, 21 QTLs, explaining more than 10% phenotypic variance, were identified as major QTLs controlling 9 morphological, 3 physiological, and 2 biochemical traits. Interestingly, several QTLs governing distinct parameters were found to be colocalized, suggesting either a profound correlation between the QTLs regulating these traits or their significant genomic proximity. In addition to the QTLs, we also identified 368380 SSR loci within the identified QTL regions, dinucleotides being the most abundant type (211,381). These findings provide valuable insights into the genetics of heat tolerance in hot peppers. The identified QTLs and SSR markers offer opportunities to develop heat-tolerant varieties, ensuring better crop performance under high-temperature conditions.

## Introduction

Hot pepper is a widely cultivated vegetable crop belonging to the Solanaceae family also known as the night shade family. It was originated from the wild and weedy species *Capsicum annuum var minimum* indigenous to Mexico, southern Peru, and Bolivian region of Latin America (Villalon, 1981). It is a diploid species with 2n=2x=24 (X=12) and genome size of ~ 3.5 Gb, with 75 to 80% of the genome composed of repetitive elements (Saisupriya and Saidaiah, 2021). India is the largest producer, consumer and exporter of the hot pepper in the world (Chilli outlook, 2021) with a production of 4.50 million tons of green chilli from an area of 0.418 million ha with a national average productivity of 10.7 t/ha (MOAFW, 2021). The escalating carbon dioxide and other greenhouse gas emissions as a result of relentless anthropogenic and industrial activities cause serious repercussions on our planet’s climatic equilibrium. These emissions contribute to the trapping of long wave radiations reflected back from the earth’s surface in the atmosphere consequently ushering in an insidious upsurge in global temperatures. It is an unsettling reality that between 1800 and 2012, the average surface temperature of earth was increased by 0.85°C (IPCC, 2018). Projections foretell a further ascent of 1.5°C by 2040 and a staggering 2°C by 2050 (IPCC, 2021).The maximum temperature of plains and hills exceeding 40°C and 30°C respectively is considered as heat wave. As carbon emissions continue to rise, heat waves in India are expected to last 25 times longer by 2036-65 (G20 climate risk atlas, 2021). Hot pepper is cultivated both in tropical and subtropical regions of the world up to an altitude of 600 meters above mean sea level (Gopalakrishnan, 2007). The ideal temperature range for its cultivation is between 20°C and 30°C (Gopalakrishnan, 2007). High temperature is a major abiotic stress factor which significantly affects hot pepper production. Increased flower abscission is reported when the day temperature ranges from 32°C to 38°C, and crop failure in fruit setting occurs at temperatures above 40°C (Srivastava et al., 2022). Such high temperatures adversely affect the normal physiological and metabolic functions within the plants, so plants have evolved unique mechanisms to withstand high-temperature conditions. These mechanisms include traits such as leaf area, canopy temperature depression (CTD), stomatal density, pollen viability, stigma and ovary health, membrane stability, photosystem II stability, transpiration, and activity of antioxidant enzymes. However, these component traits of heat stress tolerance are dispersed among different lines and varieties, each exhibiting varying degrees of resilience. Unfortunately, many popular varieties cultivated over vast areas have been significantly affected by high-temperature conditions, resulting in substantial reduction in pepper yields. Understanding the genetic basis of pepper heat tolerance is essential for devising strategies to combat heat stress as well as for developing heat tolerant varieties. Heat tolerance is a complex phenomenon that is known to be controlled polygenically by set of genes referred as Quantitative trait loci (QTL).The identification of QTLs for heat tolerance is carried out through linkage mapping by developing the dedicated mapping populations as well as by genome wide association analysis of natural populations. Several QTLs governing heat tolerance have been identified in various crops (Jha et al., 2014). These QTLs have been subsequently transferred into the genetic background of elite varieties lacking heat tolerance resulting in heat tolerant varieties with superior agronomic performance. Though few heat-tolerant genotypes have been identified in hot pepper (Usman et al., 2014; Dahal et al., 2006; Srivastava et al., 2022), however, currently no information is available regarding the QTLs responsible for heat tolerance in hot pepper. Our group has been actively engaged in the evaluation of chilli germplasm for identification of heat tolerant genotypes and through our research efforts we have successfully identified few heat tolerant lines including DLS-161-1 (Srivastava et al, 2020; Srivastava et al, 2022). The heat tolerant line DLS-161-1 used in the present study has been registered with Indigenous Collection (IC) number IC0646850 and registration number INGR22158 with National Bureau of Plant Genetic Resources, New Delhi, India.

The objective of the present study was to dissect the genetic architecture underlying heat tolerance in hot pepper for which a biparental F_2_ mapping population was developed by crossing the heat tolerant (DLS-161-1) and heat susceptible (DChBL-240) genotypes which exhibit contrasting phenotypic differences for heat tolerance.

## Materials and methods

### Plant materials and treatment conditions

The two genotypes of hot pepper which have performed contrastingly for heat tolerance consistently for four generations (**Supplementary Figure S1**), were crossed using DLS-161-1 (heat tolerant) as maternal parent (P1) and DChBL-240 (heat sensitive) as a male parent (P2). The morphological, physiological, and biochemical traits of five randomly selected plants per replication were recorded for each parent and data were recorded in three replications and mean value was calculated for the parents.

F_2_ seeds were collected from multiple fruits of a single F_1_ individual and 91 F_2_ individuals along with both parents were sown during February, 2022 in plastic protrays (96 celled, 54 x27 cm in size) filled with perlite, coco-peat and vermiculate (1:2:1) and the seedlings were transplanted 35 days after sowing in polyhouse conditions. The maximum (day temperature) and minimum (night temperature) temperature during the crop growing period (March-July) ranged from 24 to 46.5°C and 8 to 30.5°C respectively.

### Phenotyping

Both the parents and F_2_ individuals were evaluated under heat stress. Seeds of the test plants were sown on February, 2022. Observations were recorded on 14 morphological, 7 physiological and 4 biochemical traits under high temperature condition from April to July, 2022 during which daily maximum temperature of 31 to 46.5°C and a minimum temperature of 15.4 to 30.5°C was observed (**Supplementary Figure S2**).

#### Morphological traits

The data was recorded on morphological traits. Plant height (PH) was directly measured in centimeters (cm) using a scale, while the number of primary branches (PB) and number of fruits per plant (FPP) were counted. Traits such as average fruit length (AFL) and average fruit weight (AFW) were determined by measuring ten randomly selected fruits, with length recorded in centimeters (cm) and weight in grams (g). The number of healthy seeds per fruit (NS) was recorded from three randomly selected fruits and averaged. Electronic weighing balance was used to measure fruit yield per plant (FYP), fresh biomass weight (FBW) and 100 seed weight (HSW) which were expressed in grams (g). Leaf parameters, including leaf area (LA) in square centimeters (cm^2^), leaf length (LL) in centimeters (cm), leaf width (LW) in centimeters (cm), leaf perimeter (LP) in centimeters (cm), and leaf aspect ratio (AR), were recorded from the top ten leaves of each plant using WinFOLIA basic software (Regent Instruments, Inc. Canada), and the values were averaged.

#### Physiological parameters

The physiological traits like canopy temperature (CT) and canopy temperature depression (CTD) were measured in degrees Celsius (°C) using a handheld infrared thermometer (Fluke-62-Max). The normalized difference vegetation index (NDVI) which is dimensionless and is an indicator of ground cover and plant greenness, was recorded using a green seeker (Handheld-505). Stomatal density (SD) was measured by examining imprints of the lower leaf surface under a light microscope with Magvision Imaging tool (Magnus Opto Systems, India) and expressed as number of stomata mm^-2^. Pollen viability (PV) was determined as a percentage (%) using the acetocarmine test (2.5%). Membrane stability index (MSI) was measured as a percentage (%) using a conductivity meter following the procedure described by Deshmukh et al. (1991). Net photosynthetic rate (NPR) was measured using the LI-6400 portable photosynthesis system from LI-COR (Lincoln, Nebraska) on physiologically mature leaves during a sunny morning between 9 a.m. and 11 a.m. and expressed in micromoles of carbon dioxide per square meter per second (μmol CO_2_ m^-²^s^-1^).

#### Biochemical parameters

The relative chlorophyll content (RCC) of the topmost recently matured leaves was measured with CCM-200 plus chlorophyll meter (Opti-Sciences, Inc., Hudson, USA), and the activity of antioxidant enzymes such as guaiacol peroxidase (GPX), catalase (CAT), superoxide dismutase (SOD) were recorded by following the protocols of Chance and Maehly (1955); Aebi (1984) and Dhindsa et al. (1981) respectively.

### Statistical analysis

The mean, range, skewness, kurtosis and frequency distribution of all the above traits were analyzed and the histograms representing frequency distribution of F_2_ population were derived using IBM SPSS v.26.

Correlation analysis was performed among different morphological, physiological, and biochemical traits to understand the relationships and dependencies between them under high temperature conditions and correlation matrix was plotted using metan package of R package (Olivoto and Lúcio, 2020).

### Genotyping and construction of linkage maps

#### Genomic DNA isolation, purification, and quantification

The isolation and purification of genomic DNA of both the parents and 91 F_2_ individuals was carried out by following CTAB method with slight modifications (Murray and Thompson, 1980). Both Agarose gel electrophoresis (0.8%) and Nanodrop 2000 spectrophotometer (Thermo Fisher Scientific) was used to assess the quality and quantity of DNA samples. For sequencing purpose, the DNA was further purified using Qiagen DNAeasy Plant mini kit. Only DNA with A_260_/A_280_ ratio of ≥1.8 was considered good for further sequencing.

#### Library preparation, genotyping, variant calling and construction of linkage maps

For each sample, a total of 300 nanograms of DNA (6 μl in total, with a concentration of 50 ng/μl) was subjected to double digestion using the EcoRI-HF (rare-cutting) and MseI (frequent-cutting) enzymes (New England BioLabs, Ipswich, MA). The DNA was digested for four hours at 37°C, followed by heat deactivation of the enzymes at 65°C for 10 minutes. The resulting digested DNA fragments were ligated with the EcoRI-specific P1 adapter and the MseI-specific P2 adapter, using the T4 ligase enzyme (New England BioLabs, Ipswich, MA). The ligation reaction was performed by incubating the mixture overnight (>12 hours) at room temperature (approximately 21°C), followed by heat deactivation of the enzyme at 65°C for 10 minutes. To remove unincorporated adapters and small DNA fragments (<300 base pairs), the ligation reactions were purified using 0.8X volume of Agencourt AMPure XP SPRI magnetic beads (Beckman Coulter). A unique combination of the dual-indexed barcodes was attached to purified fragments with 14 cycles of PCR. The indexed PCR products were then pooled in equal volumes, and fragments with sizes ranging from 300 to 700 base pairs were selected using Agencourt AMPure XP SPRI magnetic beads. The final libraries were analyzed for size using a Tape Station instrument (Agilent Technologies, Santa Clara, CA) and the library concentration was determined using a Qubit™ 3 Fluorometer with the Qubit™ 1X dsDNA HS Assay Kit. The final DNA libraries were sequenced on a single lane of the Novaseq 6000 platform from Illumina^®^ Inc., San Diego, CA, USA, using V4 sequencing chemistry.

The sequencing data was obtained in FastQ format. To ensure the quality of the raw data and remove adapter contamination, the data were processed using FAST QC and Trim Galore v0.6.2. The processed reads were aligned to the reference genome of *Capsicum annum*, which was downloaded from http://peppergenome.snu.ac.kr/download.php. Variant calling was performed using the GATK pipeline v3.6. The resulting variants were filtered, removing indels with vcftools v0.1.16, and only biallelic SNPs were retained. These SNPs were further filtered based on parent information and a minor allele frequency (MAF) threshold of 5%. QTL-ICIMapping v4.2.53 (Meng et al., 2015) was used to discard markers that lacked polymorphism in the progenies or failed the chi-square test (with marker segregation ratio of 1:2:1) at a significance level of P=0.01. Finally, JoinMap v4.1 was employed to map and group the markers, with a LOD threshold of 3.0. The genetic distance between the markers was estimated using the Kosambi mapping function.

#### QTL mapping

The identification of QTLs for different morphological, physiological and biochemical traits studied under heat stress was performed using composite interval mapping with ICIM function of QTL ICI Mapping 4.2.53 tool (Meng et al., 2015). 1000 permutations were used to determine the LOD threshold. A QTL with a LOD threshold of 2.5 was considered a significant QTL. Genetic maps for locating the QTLs were prepared by using MapChart v2.32 (Voorrips, 2002) and observations regarding QTL name, chromosome number, left and right CI (cM), left and right coordinates, LOD, PVE (%), additive effect and QTL size (Mb) were recorded.

## Results

### Heat tolerance in F_2_ population

**Figure 1:**
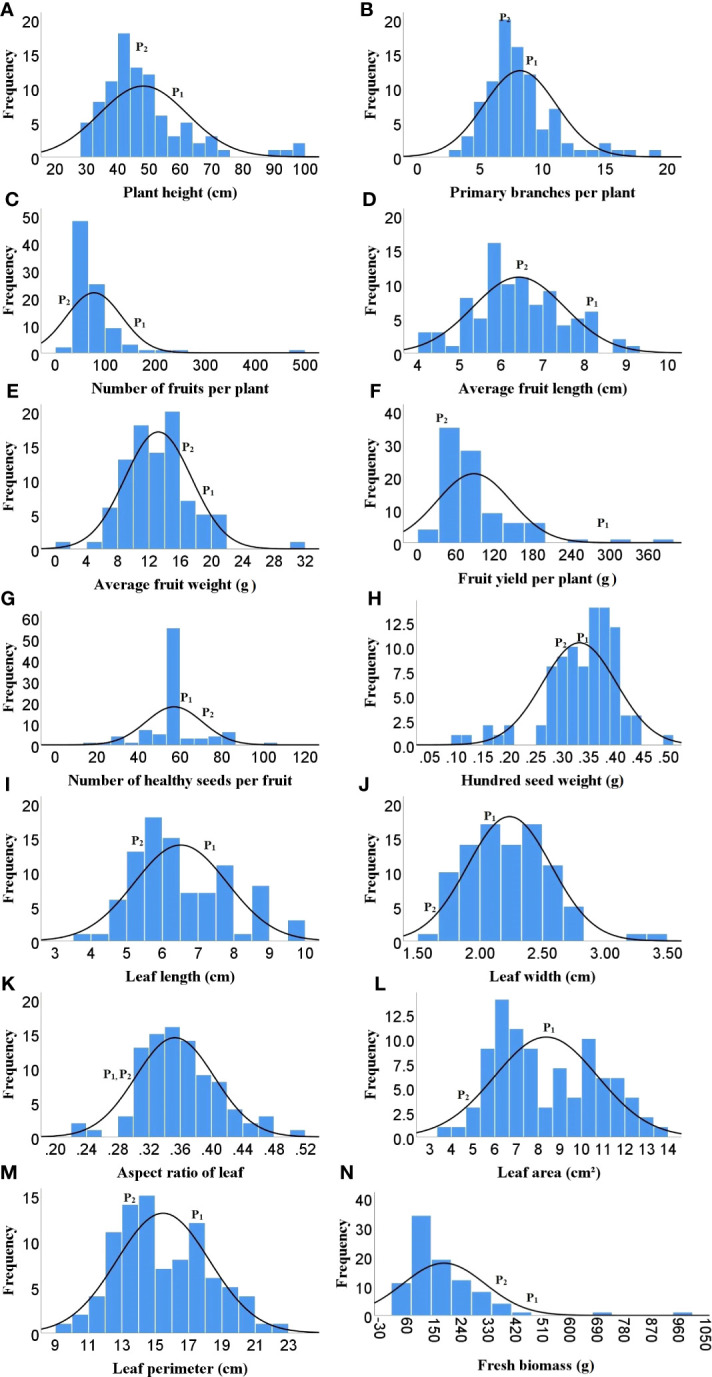
Frequency distribution of F_2_ population for different morphological traits. **(A)** Plant height, **(B)** Number of primary branches per plant. **(C)** Number of fruits per plant, **(D)** Average fruit length, **(E)** Avg fruit weight, **(F)** Fruit yield per plant, **(G)** Number of healthy seeds per fruit, **(H)** Hundred seed weight, **(I)** Leaf length, **(J)** Leaf width, **(K)** Aspect ratio, **(L)** Leaf area, **(M)** Leaf perimeter, **(N)** Fresh biomass weight, ( P1)- DLS-161-1, (P2)- DChBL-240.

**Figure 2:**
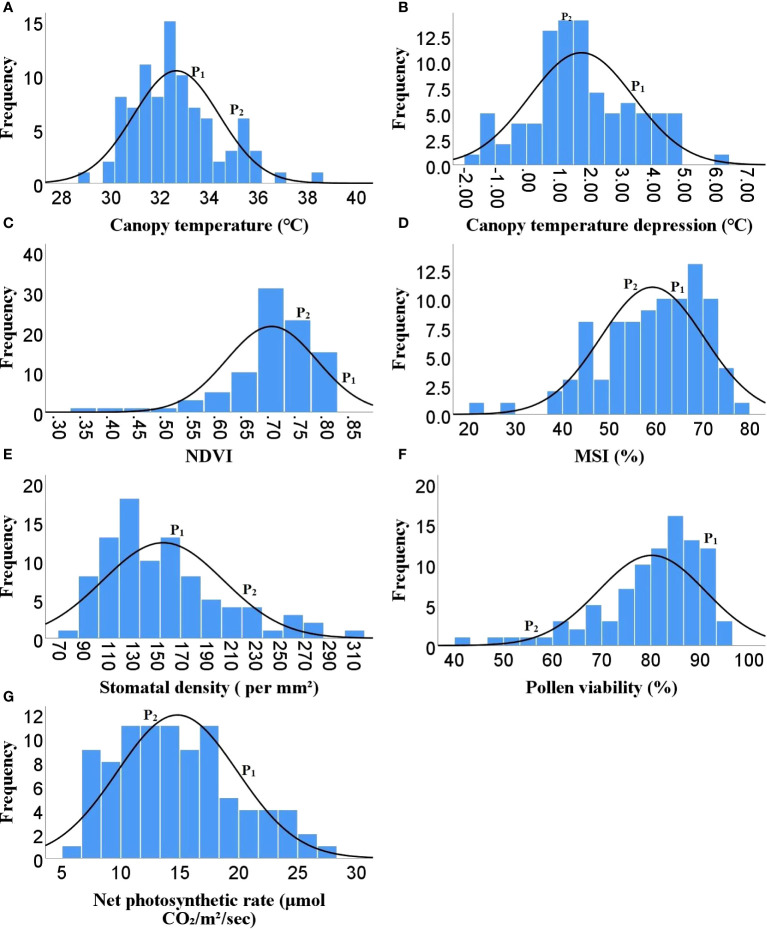
Frequency distribution of F_2_ population for different physiological traits **(A)** Canopy temperature, **(B)** Canopy temperature depression, **(C)** NDVI, **(D)** MSI, **(E)** Stomatal density, **(F)** Pollen viability, **(G)** Net photosynthetic rate, ( P1)- DLS-161-1, (P2) DChBL-240.

**Figure 3:**
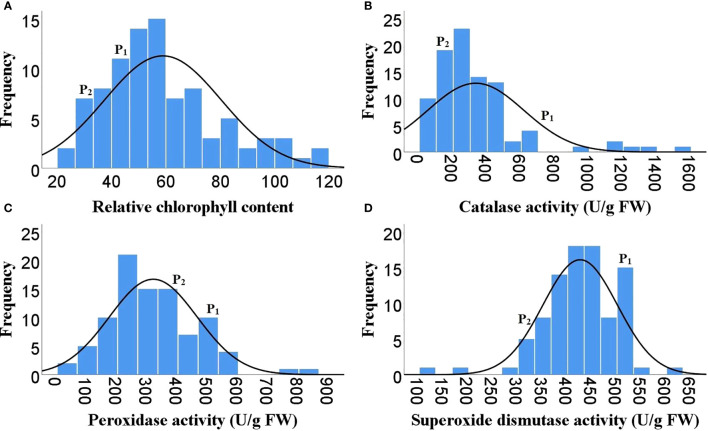
Frequency distribution of F_2_ population for different biochemical traits. **(A)** Relative chlorophyll content, **(B)** Catalase activity, **(C)** Guaiacol peroxidase activity, **(D)** Superoxide dismutase activity, ( P1)- DLS-161-1, (P2) DChBL-240.

As Heat tolerance is a complex trait and can be estimated indirectly through yield and yield contributing traits under heat-stress, therefore the phenotyping was done for twenty-five different traits governing morphological, physiological and biochemical responses of hot pepper under heat stress. A wide range of variability was recorded among the F_2_ population for all the studied traits (**Table 1**). The plant height in the F_2_ population ranged from 29.1 to 96.5 cm, while number of primary branches per plant varied from 3 to 19. Similarly the 91 F_2_ individuals produced 22 to 470 fruits per plant with a total fruit yield of 13.85 to 377.6 g. The average fruit length ranged from 4.28 to 9.32 cm and the F_2_ progenies produced 19.67 to 104.7 healthy seeds per fruit and fresh biomass of 38 to 977 g (**Table 1** and **Figure 1**). The physiological traits such as canopy temperature in the population ranged from 28.90 to 38.5°C, while CTD varied from -1.90 to 6.30°C, the MSI ranged between 22.14 to 76.69%, while stomatal density and pollen viability ranged between 74.85 to 302.06 per mm^2^ and 40.47 to 95.46% respectively (**Table 1** and **Figure 2**). Furthermore, the F_2_ individuals exhibited net photosynthetic rates of 6.45 to 27.69 μmol CO_2_ m^-^²s^-1^ (**Table 1** and **Figure 2**). The activities of catalase, guaiacol peroxidase and superoxide dismutase among the F_2_ progenies varied between 50 to 1571.4, 34.57 to 826.88 and 111.11 to 600 U/g fresh weight respectively, while the RCC ranged from 23 to 115.6 CCI (**Table 1** and **Figure 3**).

**Table 1:** Descriptive statistics of the two parental lines and F_2_ population (DLS-161-1× DChBL-240) under heat stress condition.

Traits	Parents		F_2_ population				
	DLS-161-1	DChBL-240	Mean	Minimum	Maximum	Skewness	Kurtosis
Plant height (cm)	60	48.53	48.18	29.1	96.5	1.62	3.08
No of primary branches per plant	9.67	7.33	8.21	3	19	1.33	2.47
No of fruits per plant	180.33	15.00	78	22	470	4.6	29
Average fruit length (cm)	8.40	6.89	6.42	4.28	9.32	0.25	-0.17
Average fruit weight (g)	19.02	17.90	13.34	5.76	30.31	0.90	2.40
Fruit yield per plant (g)	269.06	49.87	88.26	13.85	377.6	2.417	8.016
No of healthy seeds per fruit	65.33	75.00	57.07	19.67	104.7	0.210	2.258
100 seed weight (g)	0.33	0.30	0.33	0.1	0.49	-1.2	2.1
Leaf length (cm)	7.69	5.75	6.51	3.74	9.62	0.52	-0.33
Leaf width (cm)	2.10	1.60	2.24	1.62	3.43	0.68	1.03
Aspect ratio	0.28	0.28	0.35	0.23	0.5	0.30	0.46
Leaf area (cm^2^)	8.81	4.93	8.37	3.72	13.89	0.31	-0.95
Leaf perimeter (cm)	17.82	13.66	15.47	9.08	22.99	0.37	-0.34
Fresh biomass (g)	490.67	362.33	176.3	38	977	3.119	14.73
Canopy temperature (°C)	33.09	35.53	32.66	28.90	38.50	0.70	0.57
Canopy temperature depression (°C)	3.34	1.09	1.61	-1.90	6.30	0.29	-0.11
NDVI	0.84	0.76	0.69	0.34	0.8	-1.75	4.24
MSI (%)	65.99	55.67	59.18	22.14	76.69	-0.77	0.56
Stomatal density (number of stomata mm^-2^)	166.40	229.89	152.47	74.85	302.06	1	0.56
Pollen viability (%)	92.10	53.84	80.19	40.47	95.46	-1.31	1.91
Net photosynthetic rate (μmol CO_2_/m^2^/s)	20.73	14.27	14.8	6.45	27.69	0.45	-0.44
Relative chlorophyll content (CCI)	46.47	34.90	58.88	23	115.6	0.77	0.12
Catalase activity (U/gm FW)	711.90	195.24	332.42	50	1571.4	2.38	6.51
GPX activity (U/gm FW)	564.99	404.68	318.04	34.57	826.88	0.74	0.98
SOD activity (U/gm FW)	520.79	307.92	425.28	111.11	600	-1.05	3.10

The skewness values in the F_2_ population for number of fruits per plant (4.6), fruit yield per plant (2.41) and catalase activity (2.38) were positive and relatively high, as there were few plants with extreme phenotypic value (**Table 1** and **Figures 1**, **3**). Besides these the population was also positively skewed for plant height (1.62), number of primary branches per plant (1.33) as well as stomatal density (1.00), indicating that large proportion of the F_2_ progenies had relatively lower values for these traits (**Table 1** and **Figures 1**, **2**).The traits such as average fruit length (0.25), average fruit width (0.90), number of healthy seeds per fruit (0.21), leaf parameters (0.30-0.68), canopy temperature (0.70), canopy temperature depression (0.29), net photosynthetic rate (0.45), relative chlorophyll content (0.77) and GPX activity (0.74) were slightly positively skewed, suggesting that their distributions were close to being symmetric and distributions of the remaining traits were skewed somewhat to the left (**Table 1** and **Figures 1**–**3**).

### Correlation among the traits for heat tolerance

**Figure 4:**
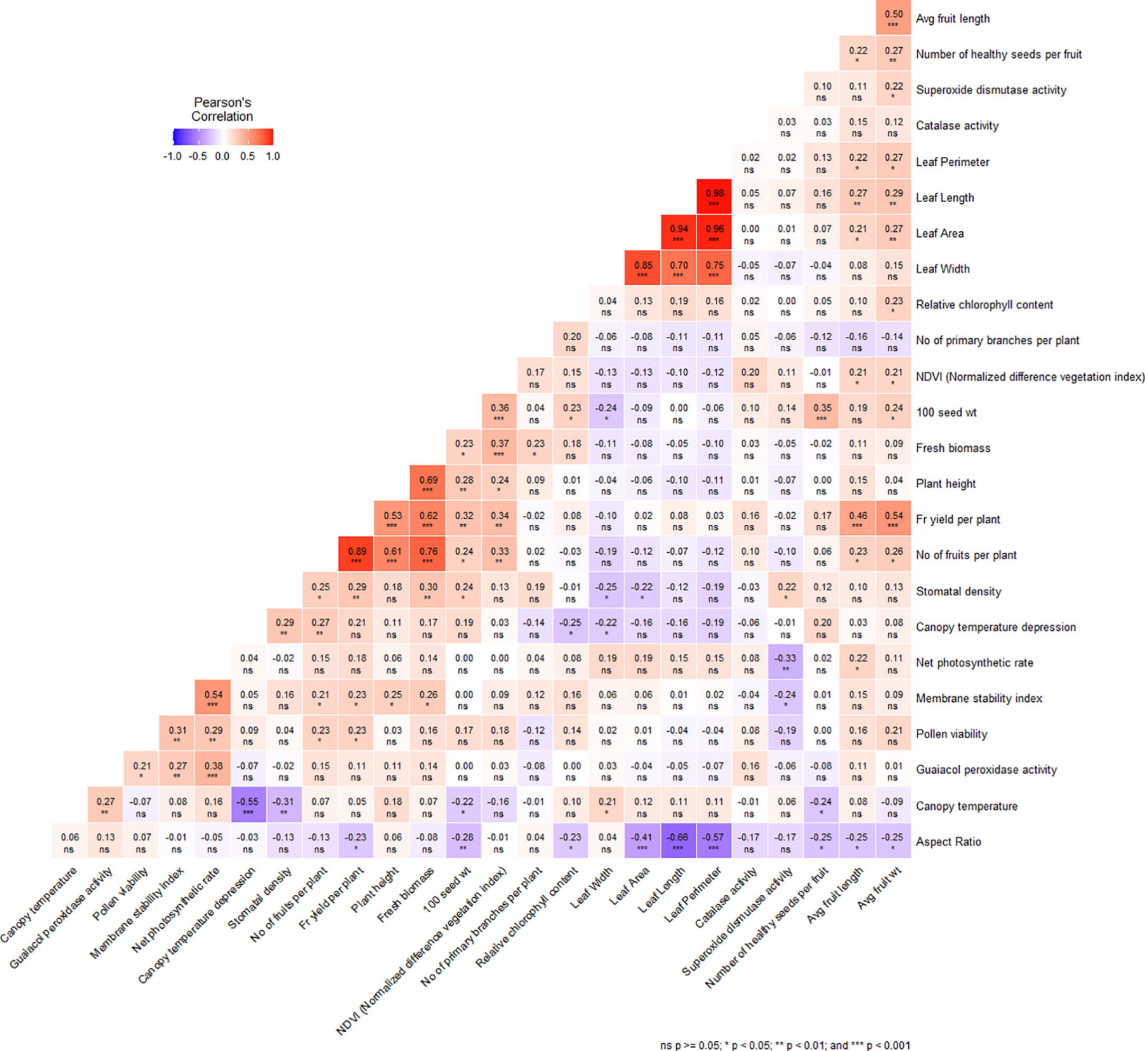
Correlation matrix plot showing the relationship between the different morphological, physiological and biochemical traits under heat stress condition. *, P ≥ 0.05, **, P ≥ 0.01, ***, P ≥ 0.001 level; ns, nonsignificant.

A comprehensive analysis of the morphological, physiological, and biochemical traits revealed noteworthy correlations among the variables investigated (**Figure 4**). Plant height exhibited a significant positive correlation with the number of fruits per plant (0.613), fruit yield per plant (0.534), hundred seed weight (0.278), NDVI (0.243), MSI (0.248). Additionally, fruit yield per plant demonstrated a strong positive association with number of fruits per plant (0.893), average fruit length (0.458), average fruit weight (0.539), number of healthy seeds per fruit (0.210), 100 seed weight (0.317), NDVI (0.339), MSI (0.229), stomatal density (0.287) and pollen viability (0.231) (**Figure 4**). Moreover, significant positive correlations were observed among various leaf parameters. For instance, leaf length displayed a robust positive correlation with leaf width (0.699), leaf area (0.935), and leaf perimeter (0.983), while exhibiting a negative correlation with leaf aspect ratio (-0.659) (**Figure 4**). In addition, both number of healthy seeds per fruit (-0.240) and 100 seed weight (-0.223) exhibited significant negative correlations with canopy temperature (**Figure 4**). Among the physiological traits, canopy temperature demonstrated a significant negative correlation with canopy temperature depression (-0.550) and stomatal density (-0.312), while displaying a positive correlation with GPX activity (**Figure 4**). Stomatal density exhibited a strong positive association with canopy temperature depression (0.288). It was also observed that MSI positively correlated with pollen viability (0.307), net photosynthetic rate (0.539), and GPX activity (0274) while RCC showed a negative correlation with canopy temperature depression (-0.246) (**Figure 4**). Additionally, SOD activity exhibited a positive correlation with stomatal density (0.220) (**Figure 4**). However, no significant correlation was observed between catalase activity and other traits examined in the present study.

### ddRAD sequencing, data processing, and linkage map construction

The paired end sequencing of 151-plex DNA libraries of both parents and F_2_ population generated 222.5629 million reads (67.215Gb) of data (**Table 2**), which was processed to remove chemical contaminants and adapters, a total of 218.93 million clean reads were retained and aligned against the pepper reference genome (**Supplementary Table S1**) and a mapping percent of 99.31 and 99.4% was observed for DLS-161-1 and DChBL-240 respectively while the mapping per cent ranged from 97.36 to 99.52% among the F_2_ progenies.

**Table 2:** Raw reads summary of the parental lines and F_2_ population.

Sample name	Number of reads	Read length (bp)	Total data in GB
P_1_ (DLS-161-1)	2088138	151	0.631
P_2_ (DChBL-240)	1353044	151	0.409
91 F_2_ population	219121675	151	66.175
**Total**	**222.5629 million**		**67.215**

**Figure 5:**
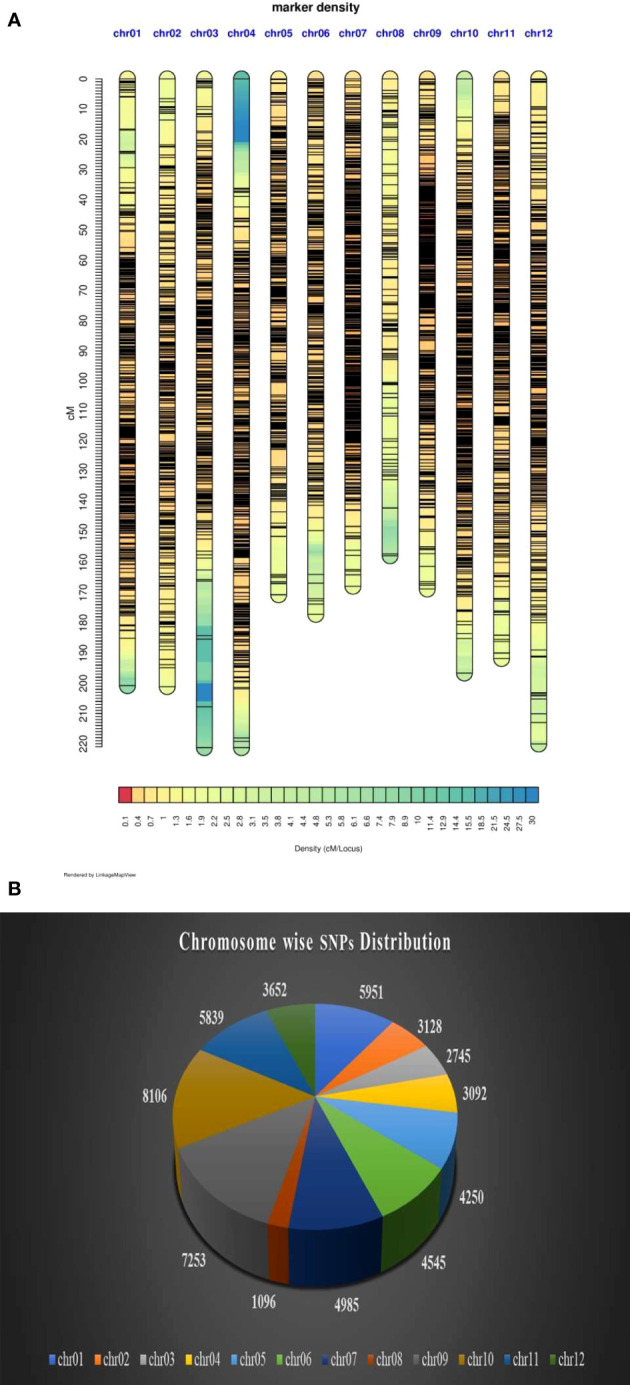
**(A)** Density plot of markers on 12 chromosomes. **(B)** Chromosome wise distribution of SNP markers.

The variant calling identified a total 41,72,807 variants, out of which 40,63,930 were SNPs and 1,08,877 were Indels. Of the total SNPs, 40,58,802 were biallelic. Parental filtering of SNPs resulted in retention of 2,59,283 SNPs, while SNP filtering especially with regard to the frequency of missing data (0.8%) and minor allele frequency (5%) retained 54,642 SNPs whose chromosome wise distribution and density plot is presented in **Figures 5** (A&B). The SNPs which did not show polymorphism in progenies and did not pass chi-square test were removed and finally only 5806 SNPs markers were grouped into 12 LGs (**Table 3**). The genetic length of the linkage groups (LG) ranged from 157.77 cM (LG8) to 221.31 cM (LG3), spanning a total map length of 2295.272 cM, with an average marker density of 0.395 cM. Among the 12 LGs, a total of 13 gaps of ≥ 10 cM were found between the markers with the maximum number of three gaps observed on LG3 (**Table 3**).

**Table 3:** Statistics of Linkage maps.

LG Name	Number of polymorphic markers	Map Length (cM)	Avg marker Distance* (cM)	Gaps (≥10cM)	Largest gap (cM)
LG 1	507	200.766	0.4	2	15.688
LG 2	389	201.132	0.52	0	8.187
LG 3	437	221.313	0.51	3	22.404
LG 4	518	221.277	0.43	2	36.246
LG 5	408	170.653	0.42	1	12.569
LG 6	372	177.172	0.48	1	14.56
LG 7	740	167.903	0.23	0	5.258
LG 8	121	157.767	1.3	1	15.128
LG 9	652	168.781	0.26	0	5.095
LG 10	620	196.6	0.32	2	12.669
LG 11	540	191.795	0.36	0	6.765
LG 12	502	220.113	0.44	1	11.793
**Total**	**5806**	**2295.272**	0.395*	**13**	

*Average marker distance.

### QTL analysis

**Figure 6:**
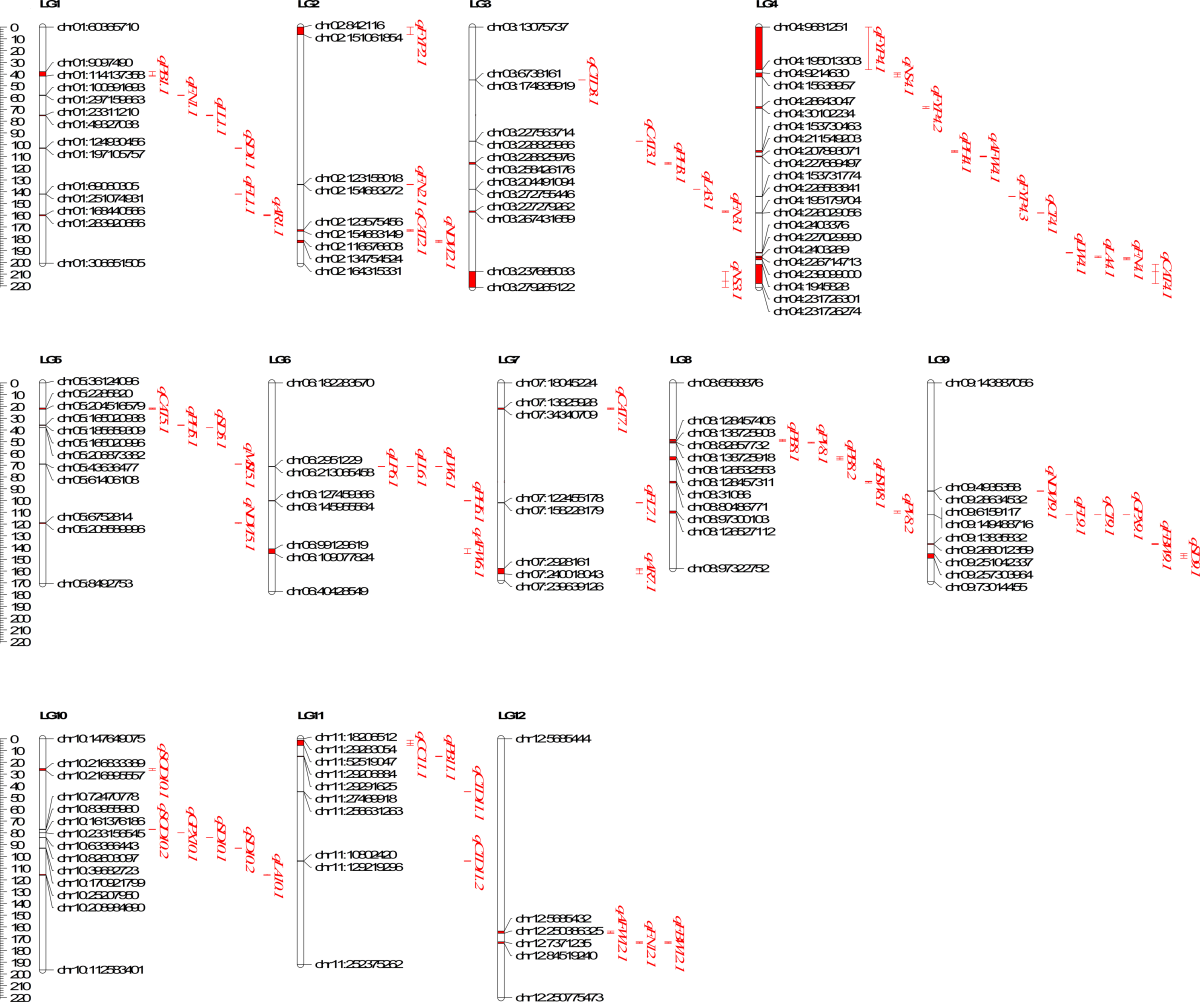
Distribution of QTLs governing different Heat tolerance traits on 12 linkage groups of *Capsicum annuum*.

The QTL analysis for different morpho-physio and biochemical traits under heat tolerance resulted in the identification of 64 QTLs for 24 out of the 25 different traits for which the phenotyping was done (**Table 4**). No QTL was detected for net photosynthetic rate. The identified QTLs were distributed across 12 linkage groups (**Table 4** and **Figure 6**). Among the 64 QTLs, those QTLs explaining >10% phenotypic variation were classified as major QTLs (**Table 5**).

**Table 4:** List of the all identified QTLs governing different morpho, physio and biochemical traits under heat stress in hot pepper.

Traits	QTL name	Chr. No.	Position (cM)	Left coordinates	Right coordinates	LOD	PVE (%)	AE	DE
Plant height	***qPH3.1***	3	116	chr03:228825976	chr03:258426176	6.71	12.16	-9.07	-9.21
	*qPH4.1*	4	106	chr04:153730463	chr04:211549203	3.30	6.70	-7.52	-7.00
	*qPH5.1*	5	36	chr05:165020938	chr05:185659309	4.34	8.42	-8.62	-0.87
	*qPH6.1*	6	100	chr06:127459366	chr06:145955564	4.11	5.08	-4.63	-5.02
No of primary branches per plant	*qPB1.1*	1	41	chr01:9097490	chr01:114137358	3.69	4.05	1.97	-1.82
	*qPB8.1*	8	49	chr08:128457406	chr08:138725903	3.63	5.57	-2.54	-2.83
	*qPB8.2*	8	64	chr08:126532553	chr08:128457311	3.16	4.51	-2.04	-1.62
	*qPB11.1*	11	15	chr11:29206884	chr11:29291625	5.23	4.98	-2.08	-2.13
No of fruits per plant	*qFN1.1*	1	58	chr01:100691693	chr01:297159663	5.76	5.54	-87.00	-87.57
	*qFN2.1*	2	134	chr02:123158018	chr02:154683272	4.97	5.56	135.36	-131.87
	*qFN3.1*	3	157	chr03:227279262	chr03:267431659	6.24	6.21	104.98	-99.65
	*qFN4.1*	4	197	chr04:226714713	chr04:239099000	4.91	6.55	123.36	-115.02
	*qFN12.1*	12	173	chr12:7371235	chr12:84519240	6.67	5.81	135.36	-122.75
Average fruit length	***qFL1.1***	1	142	chr01:69060305	chr01:251074931	2.99	10.67	0.66	-0.36
	***qFL7.1***	7	102	chr07:122455178	chr07:158228179	3.04	10.28	-0.69	0.20
	*qFL9.1*	9	112	chr09:6159117	chr09:149488716	2.65	9.99	-0.69	-0.32
Average fruit weight	***qAFW4.1***	4	110	chr04:207893071	chr04:227669497	2.53	13.25	0.70	-3.50
	***qAFW6.1***	6	145	chr06:99129619	chr06:109077824	5.53	12.74	-0.75	-3.24
	*qAFW12.1*	12	165	chr12:5685432	chr12:250386325	3.16	9.55	-0.19	3.14
Fruit yield per plant	*qFYP2.1*	2	0	chr02:842116	chr02:151061854	4.97	6.86	-3.70	40.13
	*qFYP4.1*	4	36	chr04:9681251	chr04:195013303	6.46	9.09	-9.22	-42.34
	***qFYP4.2***	4	69	chr04:28643047	chr04:30102234	11.17	19.39	3.33	66.46
	***qFYP4.3***	4	144	chr04:153731774	chr04:226583841	6.38	11.92	-40.09	-15.78
No of healthy seeds per fruit	***qNS3.1***	3	216	chr03:237685033	chr03:279265122	4.07	15.99	11.96	0.96
	*qNS4.1*	4	40	chr04:9214630	chr04:15638957	2.60	7.56	-6.13	7.82
100 seed weight	*qHSW8.1*	8	84	chr08:31086	chr08:80486771	2.85	4.37	0.04	0.06
Leaf Length	***qLL1.1***	1	75	chr01:23311210	chr01:49327038	2.98	13.17	0.59	-1.06
	***qLL6.1***	6	71	chr06:2951229	chr06:213065458	3.00	12.75	0.78	-0.82
Leaf Width	*qLW4.1*	4	192	chr04:2403376	chr04:227029990	3.52	9.16	0.15	0.27
	*qLW6.1*	6	71	chr06:2951229	chr06:213065458	3.21	9.33	0.17	-0.25
Aspect ratio	*qAR1.1*	1	160	chr01:168440566	chr01:283920656	3.21	10.00	-0.02	-0.06
	*qAR7.1*	7	159	chr07:2928161	chr07:240018043	2.73	7.00	0.01	0.04
Leaf area	***qLA3.1***	3	138	chr03:204491094	chr03:272755446	6.94	14.84	0.04	2.35
	***qLA4.1***	4	196	chr04:2403269	chr04:226714713	10.12	13.99	0.01	2.28
	***qLA10.1***	10	116	chr10:25207950	chr10:208984690	8.08	11.21	0.53	2.01
Leaf Perimeter	***qLP6.1***	6	71	chr06:2951229	chr06:213065458	3.09	13.86	1.69	-1.86
Fresh biomass	***qFBW9.1***	9	137	chr09:13835832	chr09:268012359	7.03	13.11	-308.84	-258.10
	*qFBW12.1*	12	173	chr12:7371235	chr12:84519240	2.57	9.95	217.87	-207.15
Canopy temperature	*qCT4.1*	4	158	chr04:195179704	chr04:226029056	3.21	8.70	0.88	-1.67
	*qCT9.1*	9	112	chr09:6159117	chr09:149488716	3.02	7.87	0.90	-1.93
Canopy temperature depression	*qCTD3.1*	3	45	chr03:6738161	chr03:174835919	2.85	7.44	-0.35	-1.44
	***qCTD11.1***	11	45	chr11:27469918	chr11:256631263	2.65	10.35	-0.71	1.32
	***qCTD11.2***	11	104	chr11:10802420	chr11:129219296	2.54	10.30	0.56	-1.40
NDVI	*qNDVI2.1*	2	182	chr02:116676608	chr02:134754524	4.32	9.66	0.10	0.12
	*qNDVI5.1*	5	119	chr05:6752814	chr05:208589996	2.56	7.32	-0.05	0.08
	*qNDVI9.1*	9	92	chr09:4935358	chr09:28634532	3.77	6.70	-0.06	0.04
MSI	***qMSI5.1***	5	69	chr05:43636477	chr05:61406108	3.28	17.26	5.71	8.44
Stomatal density	*qSD1.1*	1	103	chr01:124930456	chr01:197105757	4.54	8.78	-55.71	-37.12
	*qSD5.1*	5	38	chr05:165020996	chr05:206873382	4.24	9.00	-33.33	-55.74
	***qSD9.1***	9	147	chr09:251042337	chr09:257303964	6.84	12.03	32.80	-63.73
	*qSD10.1*	10	84	chr10:63366443	chr10:82603097	2.51	7.77	-35.40	-40.71
	*qSD10.2*	10	93	chr10:39682723	chr10:170921799	2.60	8.31	-41.95	-32.91
Pollen viability	*qPV8.1*	8	51	chr08:82857732	chr08:138725918	2.82	8.08	-4.08	4.52
	*qPV8.2*	8	109	chr08:97300103	chr08:126527112	3.27	8.70	-0.31	8.19
Relative chlorophyll content	***qCC11.1***	11	4	chr11:29283054	chr11:52519047	4.88	10.48	-15.14	-24.31
Catalase activity	*qCAT2.1*	2	173	chr02:123575456	chr02:154683149	7.03	5.26	357.17	-354.08
	*qCAT3.1*	3	97	chr03:227563714	chr03:228825966	9.02	5.69	-473.71	-500.14
	*qCAT4.1*	4	208	chr04:1945828	chr04:231726301	9.39	6.83	-438.58	-545.91
	*qCAT5.1*	5	22	chr05:2285820	chr05:204516579	5.09	6.56	471.87	-482.30
	*qCAT7.1*	7	22	chr07:13825928	chr07:34340709	7.99	6.25	523.37	-476.75
GPX activity	*qGPX9.1*	9	112	chr09:6159117	chr09:149488716	3.00	7.32	40.62	-159.99
	*qGPX10.1*	10	80	chr10:161376186	chr10:233156545	2.54	8.67	53.57	-142.60
SOD activity	*qSOD10.1*	10	27	chr10:216833389	chr10:216895557	3.90	9.18	-5.48	64.81
	***qSOD10.2***	10	77	chr10:72470778	chr10:83955960	2.54	10.75	-42.39	40.57

QTLs in bold are major QTLs (R^2^>10%); CI, Confidence interval ; PVE, Phenotypic variance explained; AE, Additive effect; DE, Dominance effect.

**Table 5:** List of major QTLs governing different morpho, physio and biochemical traits under heat stress in hot pepper.

Traits	QTL name	Chr. No.	Left and Right CI (cM)	Left coordinates	Right coordinates	LOD	PVE (%)	AE	DE	QTL Size (Mb)
Plant height	*qPH3.1*	3	115.5-116.5	chr03:228825976	chr03:258426176	6.71	12.16	-9.07	-9.21	29.60
Average fruit length	*qFL1.1*	1	141.5-142.5	chr01:69060305	chr01:251074931	2.99	10.67	0.66	-0.36	182.01
	*qFL7.1*	7	101.5-102.5	chr07:122455178	chr07:158228179	3.04	10.28	-0.69	0.20	35.77
Average fruit weight	*qAFW4.1*	4	109.5-110.5	chr04:207893071	chr04:227669497	2.53	13.25	0.70	-3.50	19.78
	*qAFW6.1*	6	144.5-145.5	chr06:99129619	chr06:109077824	5.53	12.74	-0.75	-3.24	9.95
Fruit yield per plant	*qFYP4.2*	4	68.5-69.5	chr04:28643047	chr04:30102234	11.17	19.39	3.33	66.46	1.46
	*qFYP4.3*	4	143.5-144.5	chr04:153731774	chr04:226583841	6.38	11.92	-40.09	-15.78	72.85
No of healthy seeds per fruit	*qNS3.1*	3	211.5-221	chr03:237685033	chr03:279265122	4.07	15.99	11.96	0.96	41.58
Leaf Length	*qLL1.1*	1	74.5-75.5	chr01:23311210	chr01:49327038	2.98	13.17	0.59	-1.06	26.02
	*qLL6.1*	6	70.5-71.5	chr06:2951229	chr06:213065458	3.00	12.75	0.78	-0.82	210.11
Leaf area	*qLA3.1*	3	137.5-138.5	chr03:204491094	chr03:272755446	6.94	14.84	0.04	2.35	68.26
	*qLA4.1*	4	195.5-196.5	chr04:2403269	chr04:226714713	10.12	13.99	0.01	2.28	224.31
	*qLA10.1*	10	115.5-116.5	chr10:25207950	chr10:208984690	8.08	11.21	0.53	2.01	183.78
Leaf perimeter	*qLP6.1*	6	70.5-71.5	chr06:2951229	chr06:213065458	3.09	13.86	1.69	-1.86	210.11
Fresh biomass	*qFBW9.1*	9	136.5-137.5	chr09:13835832	chr09:268012359	7.03	13.11	-308.84	-258.10	254.18
Canopy temperature depression	*qCTD11.1*	11	44.5-45.5	chr11:27469918	chr11:256631263	2.65	10.35	-0.71	1.32	229.16
	*qCTD11.2*	11	102.5-104.5	chr11:10802420	chr11:129219296	2.54	10.30	0.56	-1.40	118.42
MSI	*qMSI5.1*	5	68.5-69.5	chr05:43636477	chr05:61406108	3.28	17.26	5.71	8.44	17.77
Stomatal density	*qSD9.1*	9	146.5-147.5	chr09:251042337	chr09:257303964	6.84	12.03	32.80	-63.73	6.26
Relative chlorophyll content	*qCC11.1*	11	2.5-4.5	chr11:29283054	chr11:52519047	4.88	10.48	-15.14	-24.31	23.24
SOD activity	*qSOD10.2*	10	76.5-77.5	chr10:72470778	chr10:83955960	2.54	10.75	-42.39	40.57	11.49

CI, Confidence interval; LOD, logarithm of the odds; PVE, Phenotypic variance explained; AE, Additive effect; DE, Dominance effect.

A total of 21 major Quantitative Trait Loci (QTLs) were identified, of which 15 QTLs governed nine distinct morphological traits, four QTLs controlled three physiological traits and two QTLs controlled two biochemical traits. Among the morphological traits major QTLs were identified for plant height (PH), average fruit length (AFL), average fruit weight (AFW), fruit yield per plant (FYP), number of healthy seeds (NS), leaf length (LL), leaf area (LA), leaf perimeter (LP), and Fresh biomass weight (FBW) (**Table 5**); three physiological traits included canopy temperature depression (CTD), membrane stability index (MSI), and stomatal density (SD) (**Table 5**) while a single major QTL was discovered each for relative chlorophyll content and superoxide dismutase (SOD) activity (**Table 5**).

These 21 major QTLs accounted for a considerable portion of the phenotypic variance, ranging from 10.28% to 19.39% (**Table 5**). However, it is worth noting that the small population size used in the present study may have led to overestimation of the additive and dominance effects associated with some of the QTLs (Vales et al., 2005).

### Distribution of SSR repeat motifs in the identified QTLs

**Figure 7:**
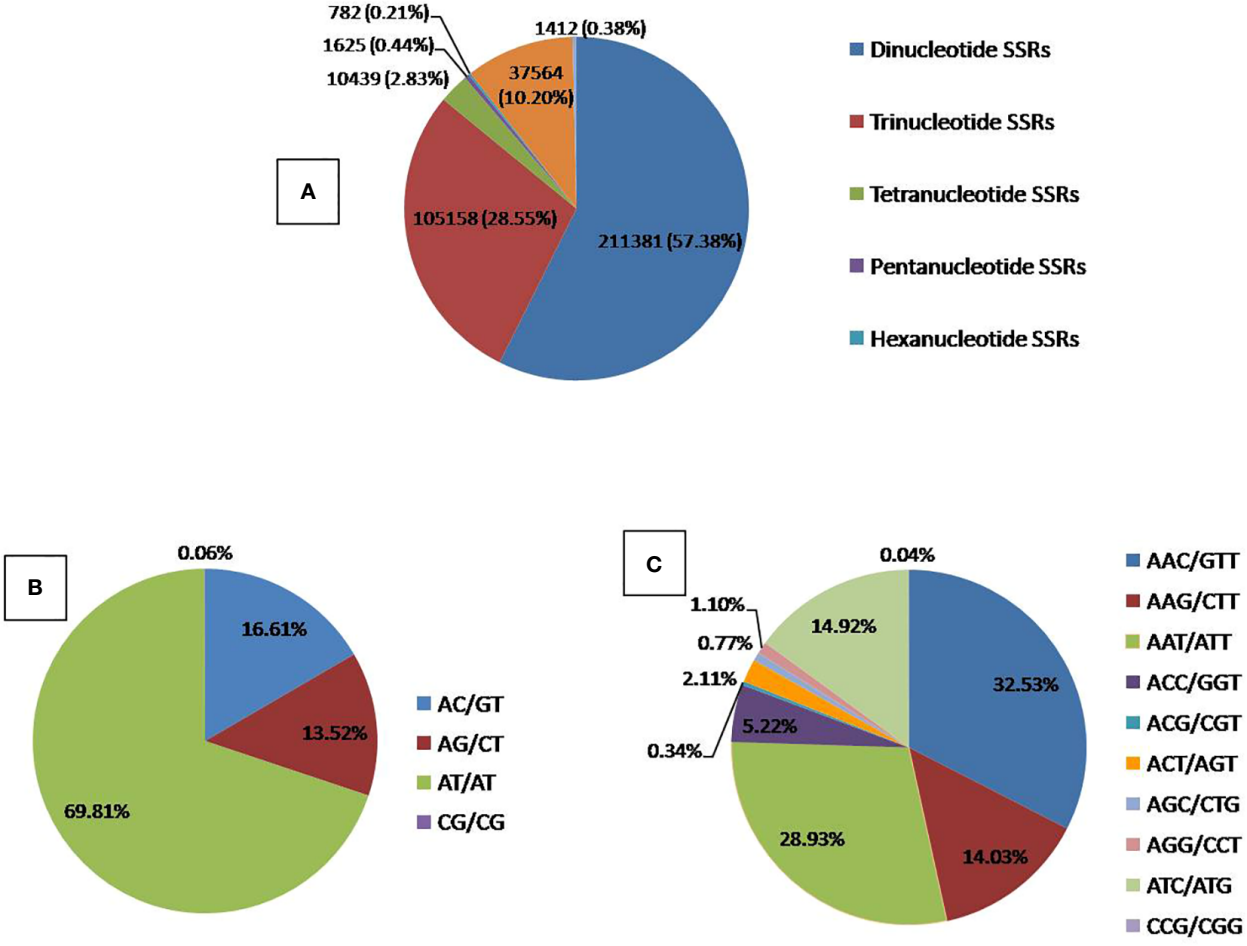
Distribution of SSR Repeat motifs in the identified QTLs. **(A)** Overall SSRs distribution, **(B)** Dinucleotide SSRs distribution, **(C)** Trinucleotide SSRs distribution.

A comprehensive analysis of our study revealed the identification of 368380 Simple Sequence Repeat (SSR) loci encompassed within 64 distinct Quantitative Trait Loci (QTLs). Among these SSRs, the most prevalent types were dinucleotide repeats, constituting a substantial proportion of 66.41% (211,381). Trinucleotide repeats followed closely, comprising 28.54% (105,158), while tetranucleotide repeats accounted for 2.83% (10,439). Pentanucleotide and hexanucleotide repeats represented smaller percentages, amounting to 0.44% (1,625) and 0.21% (782), respectively (**Supplementary Table S2** and **Figure 7**).

### QTLs identified for morphological traits under heat stress

Multiple Quantitative Trait Loci (QTLs) associated with various important traits were identified in the present study. For plant height, four QTLs were detected with LOD values ranging from 3.30 to 6.71. Among them a major QTL (*qPH3.1*) located on chromosome 3 explained 12.16% of the phenotypic variation (**Table 5**). Similarly, for the number of primary branches per plant, we identified four minor QTLs (*qPB1.1, qPB8.1, qPB8.2*, and *qPB11.1*) with LOD values ranging from 3.16 to 5.23, explaining phenotypic variation of 4.05 to 5.57% (**Table 4**). Five minor QTLs (*qFN1.1, qFN2.1, qFN3.1, qFN4.1, qFN12.1*) were identified for the number of fruits per plant with LOD values ranging from 4.91 to 6.67 and PVE ranging from 5.54 to 6.55. Moreover, we observed three QTLs for average fruit length, with LOD values ranging from 2.65 to 3.04. Among them, two major QTLs (*qFL1.1, qFL7.1*) explained 10.67 and 10.28% of the phenotypic variation respectively (**Table 4**, **5**). For average fruit weight, three genomic loci were identified, with LOD values ranging from 2.53 to 5.53. Notably, two of these loci (*qAFW4.1, qAFW6.1*) were major QTLs, explaining 13.25% and 12.74% of the phenotypic variation, respectively. Our genetic map also revealed the presence of four QTLs for fruit yield per plant, with LOD values ranging from 4.97 to 11.17. Among them, two were major QTLs (*qFYP4.2, qFYP4.3*). The QTL *qFYP4.2* exhibited a remarkably high LOD score of 11.17 and explained the highest phenotypic variation (R^2 ^= 19.39%) (**Table 5** and **Figure 6**).

In addition, two QTLs each for number of healthy seeds per fruit (*qNS3.1, qNS4.1*), leaf length (*qLL1.1, qLL6.1*), leaf width (*qLW4.1, qLW6.1*) and leaf aspect ratio (*qAR1.1, qAR7.1*) were identified (**Table 4**). Notably, *qNS3.1* for number of healthy seeds per fruit (R^2 ^= 15.99) and *qLL1.1* and *qLL6.1* for leaf length (R^2 ^= 13.17 and 12.75% respectively) were the major effect QTLs (**Table 5**). Additionally, three major QTLs, namely *qLA3.1, qLA4.1* and *qLA10.1* were identified for leaf area (R^2 ^= 14.84, 13.99 and 11.21% respectively). Another significant major QTL, *qLP6.1* contributed to leaf perimeter variation with an R^2^ value of 13.86%. Furthermore, two QTLs, *qFBW9.1* (R^2 ^= 13.11%) and *qFBW12.1* (R^2 ^= 9.95%) were found to control fresh biomass weight, while a minor QTL, *qHSW8.1*, influenced the 100 seed weight with an R^2^ value of 4.37% (**Table 4**).

### QTLs identified for physiological traits under heat stress

A total of 16 QTLs were identified for six different physiological traits under high temperature conditions (**Table 4**). Among these, two QTLs each were found for canopy temperature (*qCT4.1, qCT9.1*), and pollen viability (*qPV8.1, qPV8.2*), while a single major effect QTL, *qMSI5.1*, exerted control over MSI and accounted for a substantial proportion of phenotypic variation (17.26%). Additionally, three QTLs were identified each for canopy temperature depression (*qCTD3.1, qCTD11.1, qCTD11.2*) and NDVI (*qNDVI2.1, qNDVI5.1, qNDVI9.1*). It is worth mentioning that *qCTD11.1* and *qCTD11.2* both located on LG11 explained phenotypic variation of 10.35 and 10.30%, respectively (**Table 4** and **Figure 6**).

### QTLs identified for biochemical traits under heat stress

A single major QTL, *qCC11.1* was successfully mapped on LG11 for relative chlorophyll content explaining 10.48% of the phenotypic variation and having a LOD score of 4.88 (**Table 5**). Additionally, five minor QTLs were identified for catalase activity, with LOD values ranging from 5.09 to 9.39. These minor QTLs accounted for phenotypic variations ranging from 5.26% to 6.83% (**Table 4**). For the activity of guaiacol peroxidase, two QTLs were discovered (*qGPX9.1, qGPX10.1*) while two QTLs (*qSOD10.1, qSOD10.2*) were found to control superoxide dismutase activity (**Table 4**). Notably, *qSOD10.2* was a major effect QTL contributing 10.75% of the phenotypic variation (**Table 5**).

### Colocalization of the identified QTLs

To identify colocalizing QTLs, a comparison of the physical coordinates of each QTL was conducted. The analysis revealed several instances where QTLs shared the exact coordinates or overlapped.

On LG1, *qFN1.1* was found to colocalize with *qAR1.1* and *qSD1.1*. Additionally, *qSD1.1* overlapped with *qFL1.1*, and *qPB1.1* colocalized with *qLL1.1*. On chromosome 2, *qFN2.1* was found to colocalize with *qCAT2.1*, and *qFYP2.1* colocalized with *qNDVI2.1*. On LG3, *qFN3.1* was observed to overlap with *qPH3.1* and *qCAT3.1*. On chromosome 4, the QTLs controlling leaf area (*qLA4.1*) and leaf width (*qLW4.1*) colocalized with QTLs for fruit yield per plant (*qFYP4.1*, *qFYP4.2, qFYP4.3*). On chromosome 5, *qNDVI5.1* colocalized with *qPH5.1*, *qMSI5.1*, and *qSD5.1*. Similarly, the QTLs controlling different leaf parameters (*qLL6.1*, *qLW6.1*, *qLP6.1*) were colocalized on chromosome 6. The QTL controlling the aspect ratio of leaves (*qAR7.1*) colocalized with *qFL7.1* and *qCAT7.1* on LG7. Two QTLs controlling pollen viability (*qPV8.1* and *qPV8.2*) were found to colocalize on chromosome 8. The QTL responsible for the activity of GPX (*qGPX9.1*) colocalized with *qCT9.1* and *qFL9.1* on LG9. Additionally, the QTLs for stomatal density (*qSD10.1*, *qSD10.2*) were colocalized with each other, and the genomic region governing SOD activity (*qSOD10.2*) colocalized with *qSD10.2*. Furthermore, both *qCTD11.1* and *qCTD11.2* (associated with CTD) colocalized with *qPB11.1* and *qCC11.1*. Lastly, *qAFW12.1* colocalized with *qFN12.1* on LG12. For a detailed overview, please refer to **Supplementary Table S3**.

## Discussion

High temperature is a critical determinant that profoundly impacts the cultivation of hot pepper in tropical, subtropical and arid regions. It affects both the vegetative and reproductive stages of the crop, leading to flower and fruit abscission, ultimately resulting in a significant reduction in hot pepper yield (Srivastava et al., 2022). The F_2_ population under scrutiny in this investigation exhibited an extensive spectrum of variations across various heat-related traits, aligning harmoniously with previous studies conducted in different crops (Poli et al., 2013; Xu et al., 2017; Song et al., 2020; Jha et al., 2021; Liu et al., 2021). This observation underscores the inherent quantitative nature of heat tolerance, as affirmed by the work of Farnham and Bjorkman (2011). Moreover, multiple prior studies have proposed the polygenic control of high-temperature tolerance (Jha et al., 2014), further substantiating the complexity of this phenomenon.

The significant positive correlation between plant height and the number of fruits per plant (0.6130), fruit yield per plant (0.534), hundred seed weight (0.278), and fresh biomass (0.694) indicates that better vegetative growth helps mitigate the negative effects of high temperatures on reproductive parameters. Similar positive correlations between plant height and yield under high temperatures have been reported in previous studies (Khodarahmpour, 2012; Mason and Singh, 2014). Positive correlations of the number of fruits per plant with average fruit length (0.231), average fruit weight (0.257), fruit yield per plant (0.893), and fresh biomass (0.760) are consistent with previous studies demonstrating positive associations between fruit yield and the number of fruits, fruit length, fruit weight, and plant biomass (Poudyal et al., 2018; Rajametov et al., 2021).

Leaf area exhibited positive correlations with leaf length (0.935), width (0.848), and perimeter (0.960), but a negative correlation with the leaf length-to-width ratio (aspect ratio) (-0.411), supporting the earlier suggestion by Guo et al. (2018) regarding the relationship between different leaf parameters. The significant negative correlation of leaf aspect ratio with various fruit parameters, and that of leaf area and perimeter with average fruit length and weight, observed in this study may be attributed to the relative change of photosynthetic area with leaf size (Nicotra et al., 2011). The positive associations between the number of fruits per plant and canopy temperature depression, as well as between both the number of fruits and yield with NDVI, MSI, SD, and PV, indicate that physiological processes under high temperatures play a crucial role in the reproductive success of plants. Previous studies have also suggested positive correlations between yield under heat stress and pollen viability (which enhances successful fertilization), membrane stability (which helps maintain normal cellular functions), stomatal density (higher stomatal density aids in lowering canopy temperatures through transpiration), and vegetation index (Saint Pierre et al., 2010; Akhtar et al., 2012; Lopes and Reynolds, 2012; Kumari et al., 2013; Mondal et al., 2013; Pinto et al., 2016; Xu et al., 2017). Conversely, canopy temperature is negatively correlated with hundred seed weight (-0.223) due to reduced pollination and fertilization and increased malformed seeds (Paliwal et al., 2012). Furthermore, we also found that stomatal density is correlated positively with canopy temperature depression (0.288) and negatively with canopy temperature (-0.312), leaf area (-0.223) and leaf width (-0.248). This suggests that due to reduced epidermal cell expansion, smaller leaves are expected to have higher stomatal density, which in turn increases transpirational cooling, making the plant canopy cooler and reducing heat (Beerling and Chaloner, 1993).

The negative correlation between leaf aspect ratio and relative chlorophyll content (-0.235) can be attributed to the fact that chlorophyll levels in leaves are directly proportional to their photosynthetic capacity, and narrower leaves have a smaller photosynthetic area (Nicotra et al., 2011). High temperatures often induce the production of reactive oxygen species (ROS), which can damage cells. In response, plants produce various antioxidant enzymes to scavenge these ROS, maintaining cell membrane stability for normal cellular functioning and protecting the photosynthetic apparatus and cell membrane from oxidative stress (Ogweno et al., 2008; Asthir, 2015). This was evident in the present study, as guaiacol peroxidase activity showed positive associations with MSI (0.274), pollen viability (0.208), and net photosynthetic rate (0.382).

QTL analyses based on linkage maps with limited markers often result in large confidence intervals, reducing mapping precision and efficiency. High-throughput genotyping, utilizing next-generation sequencing (NGS) platforms has been successfully used to identify QTLs associated with heat tolerance in various vegetables, such as cowpea, tomato, broccoli, and cucumber (Lucas et al., 2013; Branham et al., 2017; Xu et al., 2017; Branham and Farnham, 2019; Dong et al., 2020). In the present study, we employed Double digest restriction-site associated DNA sequencing to develop high-density genetic maps and efficiently and cost-effectively identify QTLs controlling heat-related traits under high temperature conditions.

The present study represents the first successful identification of heat tolerance-related QTLs in hot pepper (*Capsicum annuum* L.). A total of 64 QTLs associated with 24 different traits related to high temperature tolerance were discovered. Among these QTLs, four were found to control fruit yield per plant, with *qFYP4.2* exhibiting the highest LOD score of 11.17 and explaining the highest phenotypic variance (19.39%) among the identified QTLs. This QTL had a relatively short physical length of 1.46 Mb, indicating a strong likelihood of its association with fruit yield and a major impact on the overall productivity per plant. Notably, *qFYP4.2* also displayed a positive additive effect, suggesting that the allele for increased fruit yield per plant was contributed by the heat-tolerant parent (DLS-161-1). Previous studies have reported the regulation of high-temperature fruit set by multiple QTLs, with 5-6 QTLs identified in tomato (Lin et al., 2010).

The additive effect as well as dominance effects of each of the QTL identified was estimated in the study. The additive effect of a QTL refers to the combined effect of the alleles contributed by each parent, where the trait’s value is influenced by the sum of the individual effects of the alleles at that locus. Positive additive effect indicates that alleles from maternal parent enhance the trait value, whereas negative additive effect indicates that alleles from male parent enhances the trait value (Hu et al., 2021). On the other hand, the dominance effect of a quantitative trait locus (QTL) refers to the interaction between alleles at a particular locus that results in a deviation from an additive genetic model and describes how the presence of one allele can mask or override the effect of another allele at the same locus. In F_2_ populations, understanding the additive effects of quantitative trait loci (QTLs) is more important than the dominance effects as additive effects contribute to the overall genetic variance and play a major role in determining the genetic architecture of complex traits. Additive genetic variance is directly related to the response to selection, meaning that individuals with high additive genetic values can be reliably used for breeding purposes to improve a particular trait in subsequent generations. Dominance effects are more complex and their impact on breeding decisions is less predictable (Zhang et al., 2008).

We observed that average fruit weight is controlled by two major QTLs (*qAFW4.1*, *qAFW6.1*) and a single minor QTL (*qAFW12.1*), collectively explaining 35.53% of the phenotypic variation. However, *qAFW6.1* and *qAFW12.1* exhibited negative additive effects, while *qAFW4.1* displayed a positive additive effect. Earlier studies have also reported the control of fruit weight by 2-3 major QTLs with positive additive effects in tomato under heat stress conditions (Lin et al., 2010; Bineau et al., 2021). In line with previous research on bean (Vargas et al., 2021) and tomato (Lin et al., 2010), we identified five minor QTLs for the number of fruits per plant. In the case of the number of healthy seeds per fruit, a pair of QTLs (*qNS3.1* and *qNS4.1*) exerted positive and negative additive effects, respectively. These findings align with the results reported by Lin et al. (2010) in tomato, where two major QTLs controlling seed number under heat stress displayed contrasting additive effects.

These reports further support the notion of remarkable conservation in the order and sequence of orthologs in solanaceous genomes, despite minor differences and positive gene selections (Doganlar et al., 2002; Wang et al., 2008). Previous studies on common bean have identified four major QTLs with negative additive effects for 100 seed weight under heat stress (Vargas et al., 2021). Similarly, in our study, we identified a single minor QTL controlling 100 seed weight on LG8 (LOD= 2.85, R^2 ^= 4.37%).

Regarding plant height, we found that it is controlled by three minor QTLs (*qPH4.1*, *qPH5.1*, *qPH6.1*) and a major QTL (*qPH3.1*), while pollen viability is influenced by two minor QTLs (*qPV8.1*, *qPV8.2*) with negative additive effects. Earlier studies on tomato have indicated the regulation of plant height under heat stress by two QTLs located on LG2 and LG4, respectively, while pollen viability is controlled by a single major QTL (*qPV11*), explaining 36.3% of the phenotypic variance (Xu et al., 2017). In previous studies conducted on common bean, three major QTLs (located on LG5 and LG8) were reported to influence pollen viability under heat stress conditions, collectively explaining 51.61% of the phenotypic variation (Xu et al., 2017; Vargas et al., 2021). Canopy temperature depression (CTD) serves as a measure of a plant’s cooling capacity under high-temperature conditions, crucial for maintaining optimal growth and yield. In our study, we identified a minor QTL (*qCTD3.1*) with a negative additive effect on LG3, as well as two major QTLs (*qCTD11.1*, *qCTD11.2*) on LG11, one exhibiting a positive additive effect (*qCTD11.2)* and the other a negative additive effect (*qCTD11.1*). Similarly, a consistently identified QTL with a positive additive effect for CTD (*QHtctd.bhu-7B*) has been reported in three different trials involving the RIL population of wheat (Paliwal et al., 2012).

The identification of co-localized quantitative trait loci (QTLs) governing various heat-related traits represents a remarkable opportunity to gain profound insights into the intricate mechanisms underlying heat tolerance. Such co-localizations arise from the convergence of multiple crucial genes within the same genomic region, the presence of linkage disequilibrium, or the pleiotropic effects of specific genes (Kato et al., 2000; Baytar et al., 2021). Furthermore, the mapping of correlated traits to similar genetic locations is expected since they are likely controlled by common genetic factors, establishing an intriguing genetic architecture (Kato et al., 2000).

The co-localization of *qFN1.1* and *qSD1.1* on LG1 may be attributed to the significant positive correlation observed between the number of fruits and stomatal density. Similarly, the co-localization of *qFN3.1* and *qPH3.1* can be explained by the strong positive association between the number of fruits per plant and plant height. The significant correlation between NDVI and plant height is also reflected in the overlapping QTLs controlling these traits on LG5. The significant positive correlation between GPX activity and canopy temperature is further supported by the co-localization of the corresponding QTLs on LG9. Furthermore, the QTL controlling leaf area (*qLA10.1*) was found to co-localize with the QTL controlling stomatal density, and both traits displayed a significant correlation. QTLs for SOD and stomatal density also overlapped on LG10 and exhibited a significant correlation. Similarly, the strong correlation observed between the number of fruits and fresh biomass is supported by the co-localization of their respective QTLs on LG12. Several previous studies have also indicated the relationship between trait correlation and the co-localization of QTLs controlling those traits. For example, the positive correlation between the number of inflorescences and the number of flowers per inflorescence aligns with the co-localization of QTLs controlling these traits (Xu et al., 2017). Talukder et al. (2014) identified common QTLs on LG6A, 7A, and 1D for chlorophyll content, plasma membrane damage, and thylakoid membrane damage, which were strongly correlated, suggesting pleiotropic genetic influences on these traits.

The strong correlation between leaf perimeter, leaf length, and leaf width is further supported by the fact that the loci responsible for these traits are found in the same position on LG6, indicating that the same genetic factors control all three leaf parameters. However, the small additive effect of *qLA3.1* and *qLA4.1* suggests the influence of environmental conditions or other genetic factors on these traits. Additionally, our investigation uncovered a fascinating convergence of QTLs controlling average fruit length (*qFL9.1*), canopy temperature (*qCT9.1*), and GPX activity (*qGPX9.1*), as they mapped to the same genomic region on LG6. Furthermore, the overlapping QTLs governing pollen viability (*qPV8.1* and *qPV8.2*) indicate a functional interrelation between these genetic factors, collectively influencing the phenotype.

## Conclusion

Heat-related traits are complex quantitatively inherited traits that are profoundly influenced by environmental conditions, posing significant challenges in breeding for heat tolerance in hot pepper. Nonetheless, our study successfully identified 38 QTLs governing 14 distinct morphological traits, 16 QTLs regulating 6 diverse physiological traits, and 9 QTLs orchestrating the activities of three vital antioxidant enzymes. Furthermore, a solitary QTL has been identified to govern relative chlorophyll content under high temperature conditions. Notably, the QTL *qFYP4.2*, governing fruit yield per plant, exhibited the highest phenotypic variance and LOD score, while also displaying a compact genetic region. This QTL holds tremendous potential for further fine mapping and validation.

To advance the development of heat-tolerant varieties, it is crucial to conduct targeted fine mapping of the identified QTLs. This process will help narrow down the specific genomic regions responsible for heat tolerance, providing valuable insights for future breeding efforts. Moreover, the SSR/SNP markers identified within these QTLs can be leveraged for marker-assisted selection, facilitating more efficient and precise breeding strategies for heat tolerance in hot pepper.

In conclusion, this study represents a significant advancement in our understanding of the genetic architecture underlying heat-related traits in hot pepper. The comprehensive identification of QTLs, their potential for fine mapping, and the availability of molecular markers for marker-assisted selection collectively contribute to the broader goal of developing heat-tolerant pepper varieties and empowering farmers with resilient cultivars capable of withstanding the challenges posed by high temperatures.

## Data availability statement

The sequencing data generated under the study has been submitted at NCBI SRA portal under the BioProject “PRJNA982575” with url https://www.ncbi.nlm.nih.gov/search/all/?term=PRJNA982575.

## Author contributions

Conceived theme of the study and designed experiment, MM and AS. Data curation, AT, MM, AS, and RG. Investigation, AT, AS, and MM. Resources, AS, BST, MM, BS, HK and RP. Supervision, MM, AS, and PKJ. Visualization, AS, MM, and BST. Writing original draft, AT and MM. Review and editing, MM, AS, PKJ, and TKB. All authors contributed to the article and approved the submitted version.

## Supplementary material

Supplementary Figure 1 Performance of Heat tolerant (DLS-161-1) and Heat sensitive DLS-161-1 parents under heat stress conditions (July, 2022).

Supplementary Figure 2 Temperature graph of IARI, Pusa, New Delhi during recording of observations.
