# Supplementary figures and images for: Genetic analysis of heat tolerance in hot pepper: insights from comprehensive phenotyping and QTL mapping
Source: Front Plant Sci. 2023 Aug 25;14:1232800. doi: 10.3389/fpls.2023.1232800 (PMC10491018; doi:10.3389/fpls.2023.1232800)

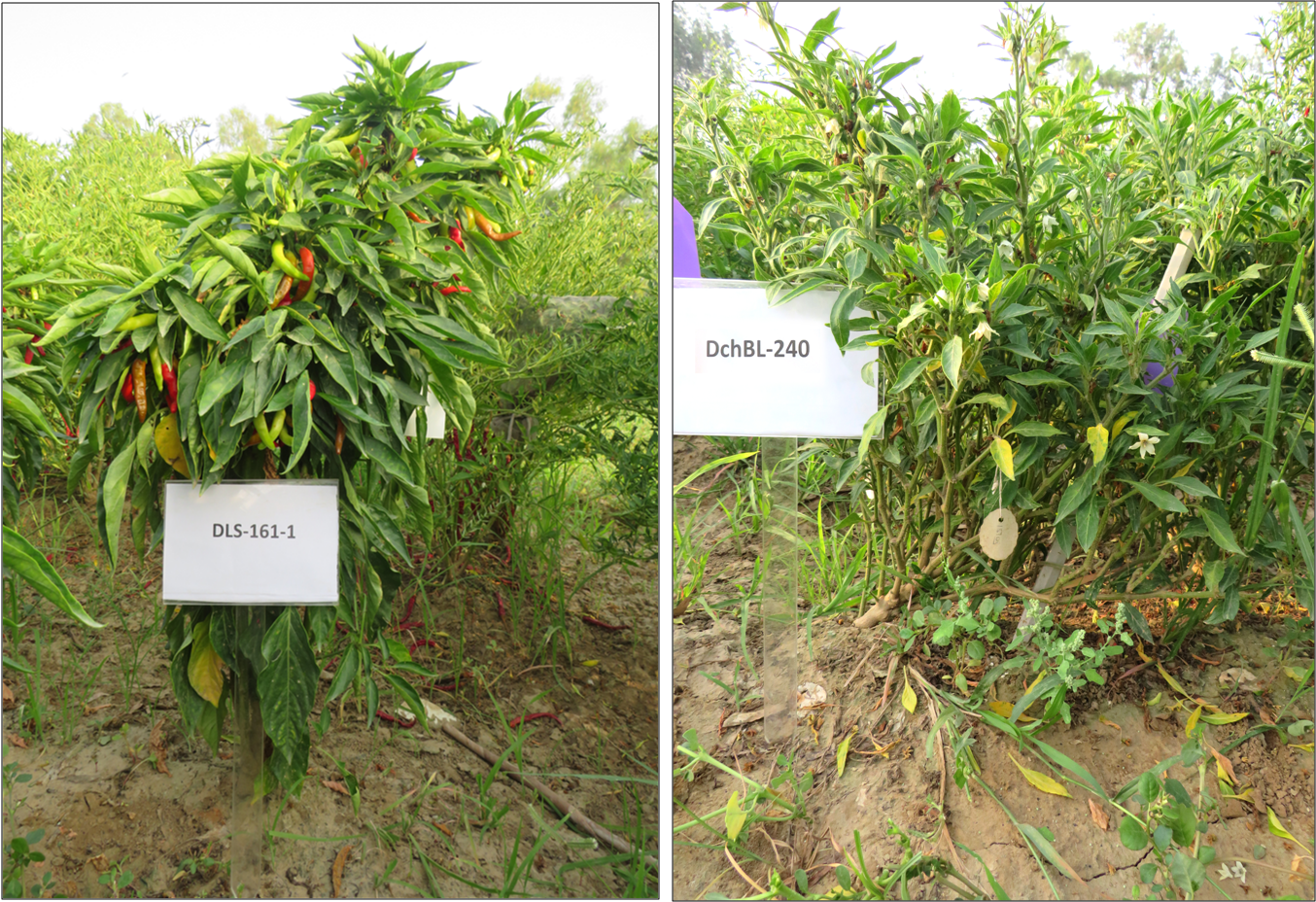

Supplement: Supplementary Figure 1 — Performance of Heat tolerant (DLS-161-1) and Heat sensitive DLS-161-1 parents under heat stress conditions (July, 2022). [file Image_1.tif]

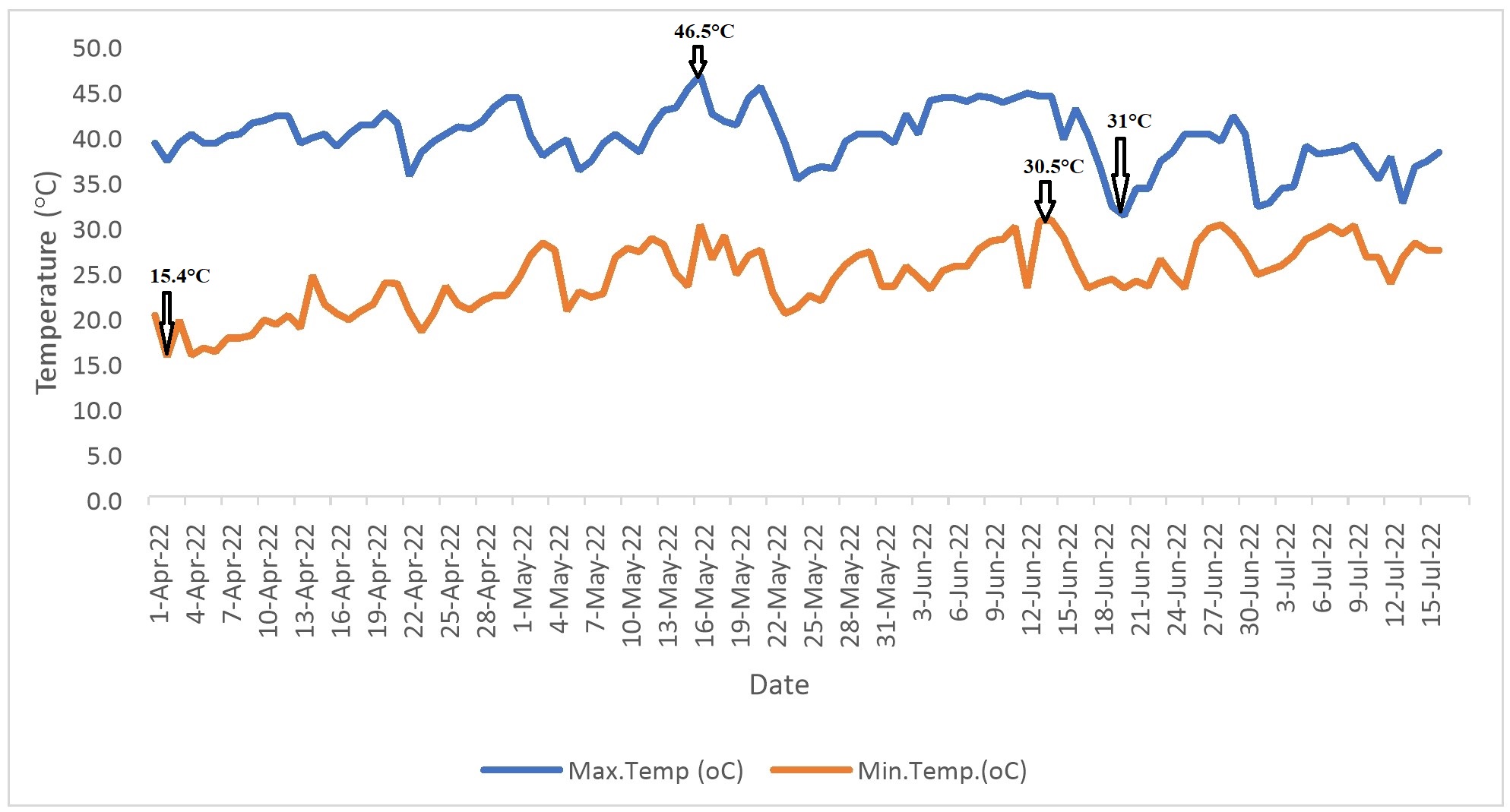

Supplement: Supplementary Figure 2 — Temperature graph of IARI, Pusa, New Delhi during recording of observations. [file Image_2.jpeg]
