# Supplementary material for: Genetic analysis of heat tolerance in hot pepper: insights from comprehensive phenotyping and QTL mapping
Source: Front Plant Sci. 2023 Aug 25;14:1232800. doi: 10.3389/fpls.2023.1232800 (PMC10491018; doi:10.3389/fpls.2023.1232800)
Supplement: Supplementary file 3 [file DataSheet_1.docx]

**Table S1: Statistics of the reference genome.**

| **Sl. No** | **Traits** | **Size** |
| --- | --- | --- |
|  | N50 (Mb) | 250.9299 |
|  | Number of Sequences | 35801 |
|  | Total Length (Gb) | 3.0639 |
|  | Min Sequence Length (bp) | 264 |
|  | Largest Sequence Length (Mb) | 309.1023 |
