# Supplementary material for: Genetic analysis of heat tolerance in hot pepper: insights from comprehensive phenotyping and QTL mapping
Source: Front Plant Sci. 2023 Aug 25;14:1232800. doi: 10.3389/fpls.2023.1232800 (PMC10491018; doi:10.3389/fpls.2023.1232800)
Supplement: Supplementary file 4 [file DataSheet_2.docx]

**Table S2 Distribution of SSR repeat motifs within the selected QTLs.**

| **Sl. No** | **QTL name** | **Dinucleotide SSRs** | **Trinucleotide SSRs** | **Tetranucleotide SSRs** | **Pentanucleotide SSRs** | **Hexanucleotide SSRs** | **Compound perfect SSRs** | **Compound Imperfect SSRs** | **Total** |
| --- | --- | --- | --- | --- | --- | --- | --- | --- | --- |
| 1 | *qPH3.1* | 1248 | 807 | 95 | 15 | 9 | 215 | 8 | 2397 |
| 2 | *qPH4.1* | 2135 | 1094 | 114 | 14 | 4 | 383 | 9 | 3753 |
| 3 | *qPH5.1* | 751 | 352 | 33 | 7 | 2 | 131 | 4 | 1280 |
| 4 | *qPH6.1* | 600 | 250 | 25 | 5 | - | 68 | 8 | 956 |
| 5 | *qPB1.1* | 3968 | 2316 | 233 | 52 | 14 | 700 | 26 | 7309 |
| 6 | *qPB8.1* | 449 | 241 | 19 | 6 | - | 63 | 5 | 783 |
| 7 | *qPB8.2* | 78 | 28 | 3 | - | - | 12 | 1 | 122 |
| 8 | *qPB11.1* | 1 | 2 | - | - | - | - | - | 3 |
| 9 | *qFN1.1* | 7225 | 3665 | 354 | 48 | 29 | 1311 | 60 | 12692 |
| 10 | *qFN12.1* | 2714 | 1640 | 163 | 23 | 9 | 506 | 16 | 5071 |
| 11 | *qFN2.1* | 1475 | 949 | 107 | 21 | 7 | 227 | 13 | 2799 |
| 12 | *qFN3.1* | 1707 | 1039 | 133 | 17 | 9 | 307 | 12 | 3224 |
| 13 | *qFN4.1* | 665 | 381 | 34 | 13 | 3 | 79 | 4 | 1179 |
| 14 | *qFL1.1* | 6679 | 3286 | 338 | 47 | 27 | 1233 | 47 | 11657 |
| 15 | *qFL7.1* | 1161 | 486 | 42 | 5 | 5 | 219 | 5 | 1923 |
| 16 | *qFL9.1* | 5172 | 2382 | 221 | 27 | 18 | 903 | 36 | 8759 |
| 17 | *qAFW12.1* | 8771 | 4627 | 433 | 71 | 33 | 1525 | 68 | 15528 |
| 18 | *qAFW4.1* | 842 | 503 | 59 | 7 | 2 | 147 | 8 | 1568 |
| 19 | *qAFW6.1* | 351 | 147 | 15 | 1 | 1 | 58 | 1 | 574 |
| 20 | *qFYP2.1* | 5681 | 3120 | 308 | 50 | 30 | 1001 | 46 | 10236 |
| 21 | *qFYP4.1* | 6212 | 2779 | 251 | 41 | 25 | 1121 | 29 | 10458 |
| 22 | *qFYP4.2* | 57 | 28 | 1 | 3 | - | 16 | - | 105 |
| 23 | *qFYP4.3* | 2780 | 1473 | 162 | 21 | 5 | 493 | 15 | 4949 |
| 24 | *qNS3.1* | 1941 | 1211 | 142 | 23 | 11 | 305 | 10 | 3643 |
| 25 | *qNS4.1* | 250 | 166 | 18 | 2 | 2 | 49 | 3 | 490 |
| 26 | *qHSW8.1* | 2872 | 1098 | 98 | 15 | 8 | 422 | 19 | 4532 |
| 27 | *qLA10.1* | 6414 | 3014 | 298 | 48 | 29 | 1128 | 33 | 10964 |
| 28 | *qLA3.1* | 2822 | 1653 | 180 | 26 | 12 | 513 | 16 | 5222 |
| 29 | *qLA4.1* | 7849 | 3725 | 355 | 53 | 28 | 1423 | 43 | 13476 |
| 30 | *qLP6.1* | 7302 | 3500 | 353 | 53 | 25 | 1332 | 54 | 12619 |
| 31 | *qLL1.1* | 999 | 583 | 52 | 12 | 4 | 157 | 4 | 1811 |
| 32 | *qLL6.1* | 7302 | 3500 | 353 | 53 | 25 | 1332 | 54 | 12619 |
| 33 | *qLW4.1* | 7860 | 3732 | 356 | 53 | 28 | 1425 | 44 | 13498 |
| 34 | *qLW6.1* | 7302 | 3500 | 353 | 53 | 25 | 1332 | 54 | 12619 |
| 35 | *qAR1.1* | 4359 | 2330 | 240 | 32 | 20 | 815 | 35 | 7831 |
| 36 | *qAR7.1* | 8440 | 4300 | 477 | 67 | 37 | 1590 | 57 | 14968 |
| 37 | *qFBW12.1* | 2714 | 1640 | 163 | 23 | 9 | 506 | 16 | 5071 |
| 38 | *qFBW9.1* | 9127 | 4275 | 416 | 54 | 38 | 1580 | 58 | 15548 |
| 39 | *qCT4.1* | 1354 | 791 | 89 | 11 | 3 | 250 | 9 | 2507 |
| 40 | *qCT9.1* | 5172 | 2382 | 221 | 27 | 18 | 903 | 36 | 8759 |
| 41 | *qCTD11.1* | 8106 | 3638 | 318 | 56 | 28 | 1472 | 65 | 13683 |
| 42 | *qCTD11.2* | 4189 | 2045 | 166 | 36 | 15 | 787 | 34 | 7272 |
| 43 | *qCTD3.1* | 5834 | 2839 | 280 | 46 | 22 | 984 | 35 | 10040 |
| 44 | *qNDVI2.1* | 689 | 433 | 47 | 4 | 3 | 123 | 10 | 1309 |
| 45 | *qNDVI5.1* | 6993 | 3262 | 319 | 55 | 14 | 1237 | 46 | 11926 |
| 46 | *qNDVI9.1* | 950 | 498 | 42 | 4 | 6 | 164 | 7 | 1671 |
| 47 | *qMSI5.1* | 564 | 285 | 25 | 12 | 3 | 106 | 5 | 1000 |
| 48 | *qSD10.1* | 612 | 268 | 23 | 1 | 6 | 81 | 2 | 993 |
| 49 | *qSD10.2* | 4507 | 1895 | 185 | 28 | 21 | 773 | 22 | 7431 |
| 50 | *qSD1.1* | 2608 | 1202 | 120 | 16 | 11 | 477 | 16 | 4450 |
| 51 | *qSD5.1* | 1500 | 811 | 93 | 18 | 4 | 302 | 9 | 2737 |
| 52 | *qSD9.1* | 234 | 139 | 15 | 3 | - | 37 | - | 428 |
| 53 | *qPV8.1* | 2069 | 979 | 103 | 19 | 5 | 341 | 17 | 3533 |
| 54 | *qPV8.2* | 1055 | 538 | 64 | 8 | 4 | 190 | 7 | 1866 |
| 55 | *qCC11.1* | 873 | 466 | 27 | 13 | 3 | 160 | 11 | 1553 |
| 56 | *qCAT2.1* | 1450 | 935 | 106 | 21 | 7 | 219 | 13 | 2751 |
| 57 | *qCAT3.1* | 51 | 17 | 5 | - | - | - | - | 92 |
| 58 | *qCAT4.1* | 8104 | 3882 | 369 | 58 | 29 | 1461 | 45 | 13948 |
| 59 | *qCAT5.1* | 7055 | 3331 | 330 | 57 | 15 | 1235 | 46 | 12069 |
| 60 | *qCAT7.1* | 741 | 459 | 73 | 19 | 1 | 177 | 4 | 1474 |
| 61 | *qGPX10.1* | 2847 | 1702 | 189 | 25 | 10 | 504 | 13 | 5290 |
| 62 | *qGPX9.1* | 5172 | 2382 | 221 | 27 | 18 | 903 | 36 | 8759 |
| 63 | *qSOD10.1* | 1 | 3 | - | - | - | 1 | - | 5 |
| 64 | *qSOD10.2* | 376 | 154 | 12 | - | 3 | 50 | 3 | 598 |
