# Supplementary material for: Genetic analysis of heat tolerance in hot pepper: insights from comprehensive phenotyping and QTL mapping
Source: Front Plant Sci. 2023 Aug 25;14:1232800. doi: 10.3389/fpls.2023.1232800 (PMC10491018; doi:10.3389/fpls.2023.1232800)
Supplement: Supplementary file 5 [file DataSheet_3.docx]

**Table S3 : List of colocalizing QTLs**

| **QTL name** | **Start coordinate (Mb)** | **Stop coordinate (Mb)** | **QTL name** | **Start coordinate (Mb)** | **Stop coordinate (Mb)** |
| --- | --- | --- | --- | --- | --- |
| *qFN1.1* | 100.69 | 297.16 | *qAR1.1* | 168.44 | 283.92 |
| *qFN1.1* | 100.69 | 297.16 | *qSD1.1* | 124.93 | 197.11 |
| *qFL1.1* | 69.06 | 251.07 | *qSD1.1* | 124.93 | 197.11 |
| *qPB1.1* | 9.10 | 114.14 | *qLL1.1* | 23.31 | 49.33 |
| *qFN2.1* | 123.16 | 154.68 | *qCAT2.1* | 123.58 | 154.68 |
| *qFYP2.1* | 0.84 | 151.06 | *qNDVI2.1* | 116.68 | 134.75 |
| *qFN3.1* | 227.28 | 267.43 | *qPH3.1* | 228.83 | 258.43 |
| *qFN3.1* | 227.28 | 267.43 | *qCAT3.1* | 227.56 | 228.83 |
| *qLA3.1* | 204.49 | 272.76 | *qFN3.1* | 227.28 | 267.43 |
| *qLA3.1* | 204.49 | 272.76 | *qPH3.1* | 228.83 | 258.43 |
| *qLA3.1* | 204.49 | 272.76 | *qCAT3.1* | 227.56 | 228.83 |
| *qFYP4.1* | 9.68 | 195.01 | *qFYP4.2* | 28.64 | 30.10 |
| *qFYP4.3* | 153.73 | 226.58 | *qCT4.1* | 195.18 | 226.03 |
| *qLA4.1* | 2.40 | 226.71 | *qFYP4.1* | 9.68 | 195.01 |
| *qLA4.1* | 2.40 | 226.71 | *qFYP4.2* | 28.64 | 30.10 |
| *qLA4.1* | 2.40 | 226.71 | *qFYP4.3* | 153.73 | 226.58 |
| *qLA4.1* | 2.40 | 226.71 | *qNS4.1* | 9.21 | 15.64 |
| *qLA4.1* | 2.40 | 226.71 | *qPH4.1* | 153.73 | 211.55 |
| *qLA4.1* | 2.40 | 226.71 | *qCT4.1* | 195.18 | 226.03 |
| *qLW4.1* | 2.40 | 227.03 | *qFYP4.1* | 9.68 | 195.01 |
| *qLW4.1* | 2.40 | 227.03 | *qFYP4.2* | 28.64 | 30.10 |
| *qLW4.1* | 2.40 | 227.03 | *qFYP4.3* | 153.73 | 226.58 |
| *qLW4.1* | 2.40 | 227.03 | *qNS4.1* | 9.21 | 15.64 |
| *qLW4.1* | 2.40 | 227.03 | *qPH4.1* | 153.73 | 211.55 |
| *qLW4.1* | 2.40 | 227.03 | *qCT4.1* | 195.18 | 226.03 |
| *qCAT4.1* | 1.95 | 231.73 | *qFYP4.1* | 9.68 | 195.01 |
| *qCAT4.1* | 1.95 | 231.73 | *qFYP4.2* | 28.64 | 30.10 |
| *qCAT4.1* | 1.95 | 231.73 | *qFYP4.3* | 153.73 | 226.58 |
| *qCAT4.1* | 1.95 | 231.73 | *qAFW4.1* | 207.89 | 227.67 |
| *qCAT4.1* | 1.95 | 231.73 | *qNS4.1* | 9.21 | 15.64 |
| *qCAT4.1* | 1.95 | 231.73 | *qPH4.1* | 153.73 | 211.55 |
| *qCAT4.1* | 1.95 | 231.73 | *qLA4.1* | 2.40 | 226.71 |
| *qCAT4.1* | 1.95 | 231.73 | *qLW4.1* | 2.40 | 227.03 |
| *qCAT4.1* | 1.95 | 231.73 | *qCT4.1* | 195.18 | 226.03 |
| *qNDVI5.1* | 6.75 | 208.59 | *qPH5.1* | 165.02 | 185.66 |
| *qNDVI5.1* | 6.75 | 208.59 | *qMSI5.1* | 43.64 | 61.41 |
| *qNDVI5.1* | 6.75 | 208.59 | *qSD5.1* | 165.02 | 206.87 |
| *qCAT5.1* | 2.29 | 204.52 | *qPH5.1* | 165.02 | 185.66 |
| *qCAT5.1* | 2.29 | 204.52 | *qMSI5.1* | 43.64 | 61.41 |
| *qLP6.1* | 2.95 | 213.07 | *qAFW6.1* | 99.13 | 109.08 |
| *qLP6.1* | 2.95 | 213.07 | *qPH6.1* | 127.46 | 145.96 |
| *qLP6.1* | 2.95 | 213.07 | *qLL6.1* | 2.95 | 213.07 |
| *qLP6.1* | 2.95 | 213.07 | *qLW6.1* | 2.95 | 213.07 |
| *qLL6.1* | 2.95 | 213.07 | *qAFW6.1* | 99.13 | 109.08 |
| *qLL6.1* | 2.95 | 213.07 | *qPH6.1* | 127.46 | 145.96 |
| *qLL6.1* | 2.95 | 213.07 | *qLP6.1* | 2.95 | 213.07 |
| *qLL6.1* | 2.95 | 213.07 | *qLW6.1* | 2.95 | 213.07 |
| *qLW6.1* | 2.95 | 213.07 | *qAFW6.1* | 99.13 | 109.08 |
| *qLW6.1* | 2.95 | 213.07 | *qPH6.1* | 127.46 | 145.96 |
| *qLW6.1* | 2.95 | 213.07 | *qLP6.1* | 2.95 | 213.07 |
| *qLW6.1* | 2.95 | 213.07 | *qLL6.1* | 2.95 | 213.07 |
| *qAR7.1* | 2.93 | 240.02 | *qFL7.1* | 122.46 | 158.23 |
| *qAR7.1* | 2.93 | 240.02 | *qCAT7.1* | 13.83 | 34.34 |
| *qPV8.1* | 82.86 | 138.73 | *qPV8.2* | 97.30 | 126.53 |
| *qPV8.1* | 82.86 | 138.73 | *qPB8.1* | 128.46 | 138.73 |
| *qPV8.1* | 82.86 | 138.73 | *qPB8.2* | 126.53 | 128.46 |
| *qFL9.1* | 6.16 | 149.49 | *qCT9.1* | 6.16 | 149.49 |
| *qFL9.1* | 6.16 | 149.49 | *qGPX9.1* | 6.16 | 149.49 |
| *qFBW9.1* | 13.84 | 268.01 | *qSD9.1* | 251.04 | 257.30 |
| *qCT9.1* | 6.16 | 149.49 | *qFL9.1* | 6.16 | 149.49 |
| *qCT9.1* | 6.16 | 149.49 | *qGPX9.1* | 6.16 | 149.49 |
| *qGPX9.1* | 6.16 | 149.49 | *qFL9.1* | 6.16 | 149.49 |
| *qGPX9.1* | 6.16 | 149.49 | *qCT9.1* | 6.16 | 149.49 |
| *qLA10.1* | 25.21 | 208.98 | *qSD10.1* | 63.37 | 82.60 |
| *qLA10.1* | 25.21 | 208.98 | *qSD10.2* | 39.68 | 170.92 |
| *qLA10.1* | 25.21 | 208.98 | *qSOD10.2* | 72.47 | 83.96 |
| *qSD10.2* | 39.68 | 170.92 | *qSD10.1* | 63.37 | 82.60 |
| *qSD10.2* | 39.68 | 170.92 | *qSOD10.2* | 72.47 | 83.96 |
| *qGPX10.1* | 161.38 | 233.16 | *qSOD10.1* | 216.83 | 216.90 |
| *qCTD11.1* | 27.47 | 256.63 | *qPB11.1* | 29.21 | 29.29 |
| *qCTD11.1* | 27.47 | 256.63 | *qCC11.1* | 29.28 | 52.52 |
| *qCTD11.2* | 10.80 | 129.22 | *qPB11.1* | 29.21 | 29.29 |
| *qCTD11.2* | 10.80 | 129.22 | *qCC11.1* | 29.28 | 52.52 |
| *qFN12.1* | 7.37 | 84.52 | *qFBW12.1* | 7.37 | 84.52 |
| *qAFW12.1* | 5.69 | 250.39 | *qFN12.1* | 7.37 | 84.52 |
| *qAFW12.1* | 5.69 | 250.39 | *qFBW12.1* | 7.37 | 84.52 |
| *qFBW12.1* | 7.37 | 84.52 | *qFN12.1* | 7.37 | 84.52 |
